# Genome-wide analysis of HECT E3 ubiquitin ligase gene family in *Solanum lycopersicum*

**DOI:** 10.1038/s41598-021-95436-2

**Published:** 2021-08-05

**Authors:** Bhaskar Sharma, Harshita Saxena, Harshita Negi

**Affiliations:** 1 School of Life and Environmental Sciences, Faculty of Science, Engineering, and Built Environment, Deakin University, Geelong, VIC 3220 Australia; 2 Structural and Molecular Biology Laboratory, Department of Biotechnology, TERI School of Advanced Studies, New Delhi, 110070 India

**Keywords:** Plant evolution, Plant hormones, Plant immunity, Plant physiology, Plant stress responses, Evolution, Plant sciences

## Abstract

The E3 ubiquitin ligases have been known to intrigue many researchers to date, due to their heterogenicity and substrate mediation for ubiquitin transfer to the protein. HECT (Homologous to the E6-AP Carboxyl Terminus) E3 ligases are spatially and temporally regulated for substrate specificity, E2 ubiquitin-conjugating enzyme interaction, and chain specificity during ubiquitylation. However, the role of the HECT E3 ubiquitin ligase in plant development and stress responses was rarely explored. We have conducted an in-silico genome-wide analysis to identify and predict the structural and functional aspects of HECT E3 ligase members in tomato. Fourteen members of HECT E3 ligases were identified and analyzed for the physicochemical parameters, phylogenetic relations, structural organizations, tissue-specific gene expression patterns, and protein interaction networks. Our comprehensive analysis revealed the HECT domain conservation throughout the gene family, close evolutionary relationship with different plant species, and active involvement of HECT E3 ubiquitin ligases in tomato plant development and stress responses. We speculate an indispensable biological significance of the HECT gene family through extensive participation in several plant cellular and molecular pathways.

## Introduction

Plant developmental and physiological processes are regulated by the synthesis and degradation of proteins, such that the plant can adapt to the environmental fluctuations while maintaining cellular homeostasis^1^. Post-translational modifications have a major role in protein degradation which limits the protein load in the cells through eradication of unwanted or abnormal proteins, and amino acids are recycled under strict and selective regulation^2,3^. In eukaryotes, twofold protein degradation is conducted via the lysosome-mediated cellular proteolytic system and ATP-dependent ubiquitin–proteasome system^4^. In the course of investigating selective proteolytic mechanisms, the ubiquitin-mediated protein degradation, non-lysosomal machinery, has grabbed much attention since their discovery by the Nobel laureates, Aaron Ciechanover, Avram Hershko, and Irwin Rose in the 1980s^5^. Ubiquitin (Ub) is a highly conserved monomeric protein, having 76 amino-acids and a molecular mass of 8 kDa, which is abundantly and ubiquitously present in the eukaryotic cells^4,6^. The modification of a protein by covalent attachment of ubiquitin is known as ubiquitination or ubiquitylation, wherein the lysine (Lys) residues in the target proteins are covalently linked to Ub^7,8^. Ubiquitylation can be sub-classified as (1) monoubiquitylation, which involves the single linkage of Lys-Ub; (2) multiple monoubiquitylation, incorporating multiple Lys-Ub linkages in the target protein; and (3) polyubiquitylation, in which many Ub molecules form a chain and link to the Lys residues in the target protein^6,8,9^.

Ubiquitin-mediated protein degradation is achieved through a proteasome, a multi-subunit, ATP-dependent protease, to form the ubiquitin–proteasome system (UPS)^10–12^. This intricate system is modulated by a three-enzyme cascade: the ubiquitin-activating enzyme (E1), which activates the Ub by forming a thioester bond; the ubiquitin-conjugating enzyme (E2), which assist Ub transfer to the target protein/substrate; and the ubiquitin-ligating enzyme (E3), which interact with E2 and the target protein to catalyze Ub transfer and formation of Ub chain. The formed assembly directs signals to the UPS for protein degradation^5,10^. The proteasome is a large protein complex made up of two subcomplexes; the 20S core particles (CP) and 19S regulatory particles (RP) at the terminal^11,13^. E3 ligases are the most critical part of the ubiquitination process because they produce protein-specific signals to UPS by selective screening of target proteins, and therefore, the E3 ligases are present in a large number with diversity^14,15^. In plants, E3 ligases not only play a crucial role in protein degradation but are also involved in various cellular and biological processes like phytohormones regulation, light response, biotic, and abiotic stress tolerance^1,9,16^. Based on their mode of action, target protein specificity, structural composition, and origin, the E3 ligases are classified into four major classes, namely, Really Interesting New Gene (RING), Homologous to E6-associated protein Carboxyl Terminus (HECT), Cullin-RING Ligase (CRL), and U-box^8,17–19^. The HECT E3 ligases are a family of proteins with a HECT domain containing approximately 350 amino acids^14,20,21^. The HECT E3 ligases have a bi-lobe structure studded with a catalytic HECT domain at the C- terminal (C-lobe) and the loosely conserved N- terminal (N-lobe). C-lobe contains a cysteine (Cys) active site that forms a transient thioester bond for ligation while the N-lobe interacts with E2 for ubiquitin transfer. N-lobe comprises extended diverse domains that contribute to the classification of HECT E3 ligases into different subfamilies^20–22^. The first HECT gene was identified in human papillomavirus (HPV) E6-associated protein (E6-AP) in 1995 and since then several studies have been conducted to identify HECT members in different organisms^23^. Several studies demonstrate organism-specific distribution and functional involvement of HECT E3 ubiquitin ligases and have identified 28 HECT members in *Homo sapiens*, 5 in *Saccharomyces cerevisiae*, 7 in *Arabidopsis thaliana*, 9 in *Chlamydomonas reinhardtii*, 12 in *Physcomitrella patens*, 12 in *Zea mays*, 8 in *Oryza sativa*, 19 in *Glycine max*, 13 in *Malus domestica,* 10 in *Brassica rapa,* 13 in *Brassica oleracea,* and 12 in *Solanum tuberosum*^17,18,22,24–28^.

*Solanum lycopersicum* belongs to the family *Solanaceae* and is recognized as a model organism for studying fruit development, stress responses, and domestication^29,30^. Globally, the farmers confront a substantial loss of tomato cultivation due to environmental stress and pathogen attacks every year^31–33^. The HECT E3 ligases are directly involved in selective protein degradation and could influence the plant responses to environmental stress and plant developmental pathways^27^. In this study, we have conducted an *in-silico* genome-wide analysis for the identification of HECT E3 ubiquitin ligases members in *S. lycopersicum*. The identification and characterization of the HECT E3 ligases can unravel the distribution, evolutionary aspects, and functional organization that would facilitate a better understanding of the ubiquitin-proteasomal degradation mechanism in the tomato plant. Further, exploring the role of E3 ubiquitin ligases in plant signaling, behavior, and survival can support the development of better strategies for crop protection and yield improvement.

## Results

### Identification and characterization of HECT gene family in tomato

A total of 14 homologous members of the HECT family in *S. lycopersicum* (Taxonomy ID: 4081) were identified by retrieving the profile HMM of the HECT domain from the Pfam database. Hidden Markov Model (HMM) profile is built using the HMMER program, used for extensive analyses which encompass probabilistic approaches by converting multiple sequence alignment to generate position-specific scorings and assess sequence similarities amongst proteins^34^. A significant e-value of 0.01 was used as cutoff along with other default parameters for sequence extraction and redundant sequences were excluded from the search. The fourteen tomato HECT members were named *SlHECT 1* to *SlHECT 14* according to their position on chromosomes 1 to 12. The analysis of the physicochemical properties (Supplementary Table S1) revealed the number of amino acids that ranged from 286 to 3757; molecular weight and pI varied from 33,008.62 to 412,777.85 and 4.65 to 8.3, respectively. All the proteins identified were stable and the index of aliphatic side chains varied from 81.57 to 96.28. The GRAVY values of all the proteins were found to be < 0, which indicates the hydrophilic nature of the proteins. Furthermore, the chromosomal location, gene size, number of introns and exons, and HECT domain information of the 14 putative candidates were evaluated (Supplementary Table S1). The HECT proteins were predicted to translocate majorly in the nucleus and cytoplasm, and five members were located in the chloroplast that suggests an active involvement of these proteins in organelle-specific signaling pathways.

### Phylogenetic analysis

**Figure 1:**
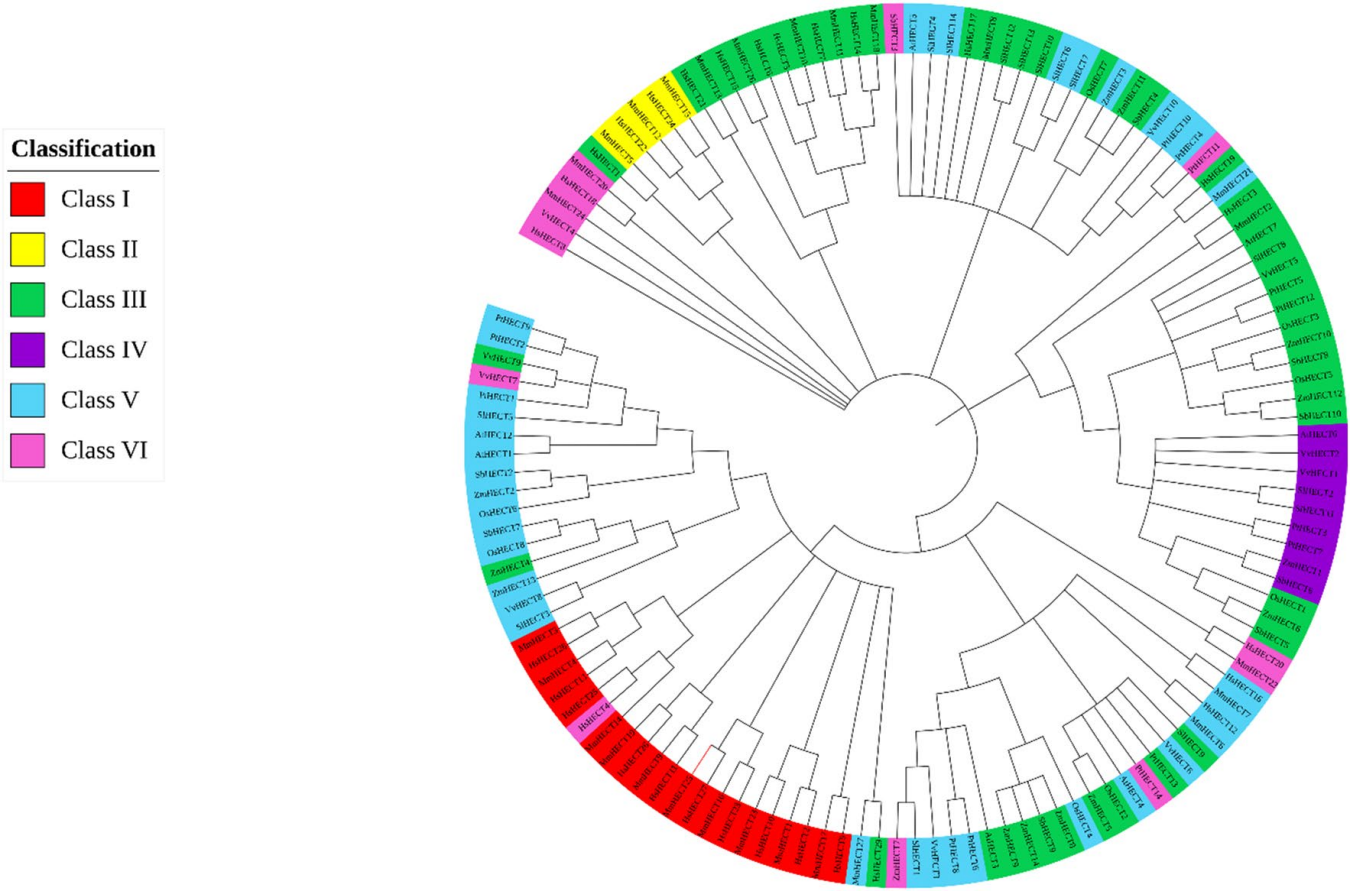
The phylogenetic tree representing the HECT E3 ubiquitin ligase members of *Solanum lycopersicum*, *Oryza sativa*, *Arabidopsis thaliana*, *Populus trichocarpa, Vitis vinifera, Sorghum bicolor, Zea mays, Mus musculus*, and *Homo sapiens* constructed by Neighbour-Joining (NJ) method with 1000 bootstrap values. The HECT gene family is sub-divided into six classes, represented by different colors (Supplementary Table S3 and Supplementary Figure S9).

To evaluate and deduce the evolutionary relatedness of the HECT gene family of *S. lycopersicum* with *Arabidopsis thaliana*, *Oryza sativa*, *Populus trichocarpa, Vitis vinifera, Sorghum bicolor, Zea mays, Mus musculus*, and *Homo sapiens*, a phylogenetic tree was constructed using the Maximum Likelihood (ML) method with 1000 bootstrap replications (Fig. 1). The identified HECT genes in all the organisms were named according to their position on the chromosomes (Supplementary Table S3). The HECT domain, in all the cases, was located at the C-terminal and the classification of the HECT gene family was based on the presence of different domains at the N- terminal (Supplementary Figure S9)^35,36^. The HECT proteins were distributed into six classes according to the presence of NEDD4 subfamily domains (Class I), HERC subfamily domains (Class II), only HECT domains (Class III), armadillo sequences (Class IV), ubiquitin-associated domains (Class V), and other domains (Class VI). We observed that 20 and 5 HECT members from Classes I and II, respectively belonged to mice and humans only. Five tomato HECT members, *SlHECT* *8, 9, 10, 12*, and *13* were classified into Class III. Many Class III members have shown similarity with the Class V members and were closely mapped. The *SlHECT 9* from Class III and *VvHECT6* from Class V shared high similarity despite the presence of different domains. Similarly, the *SlHECT* *10, 12*, and *13* of Class III were closely related to *SlHECT* *6*, and *7* of Class V. Majority of *SlHECT* members namely, *SlHECT* *1, 3, 4, 5, 6, 7*, and *14* were classified under Class V, indicating their participation in ubiquitination. Class IV was completely devoid of members from humans and mice. Class III, IV, and V members showed significant conservation that indicates the common mechanism of the divergence producing HECT members with different functional domains. We noticed a clear distance between the plant and animal HECT members. Despite the presence of the conserved HECT domain in all the sequences, the organization of the domains was quite different from that reflected in the distant evolutionary mapping. We found many common motifs in the structures of the mouse and human HECT members that were present in plants HECT members. We speculate the conservation of the HECT domains among all the HECT members and their evolution as per specific biological requirements. It is interesting to observe that genes can be selectively modified to participate in specific cellular or molecular mechanisms.

### Chromosomal localization and promoter analysis

**Figure 2:**
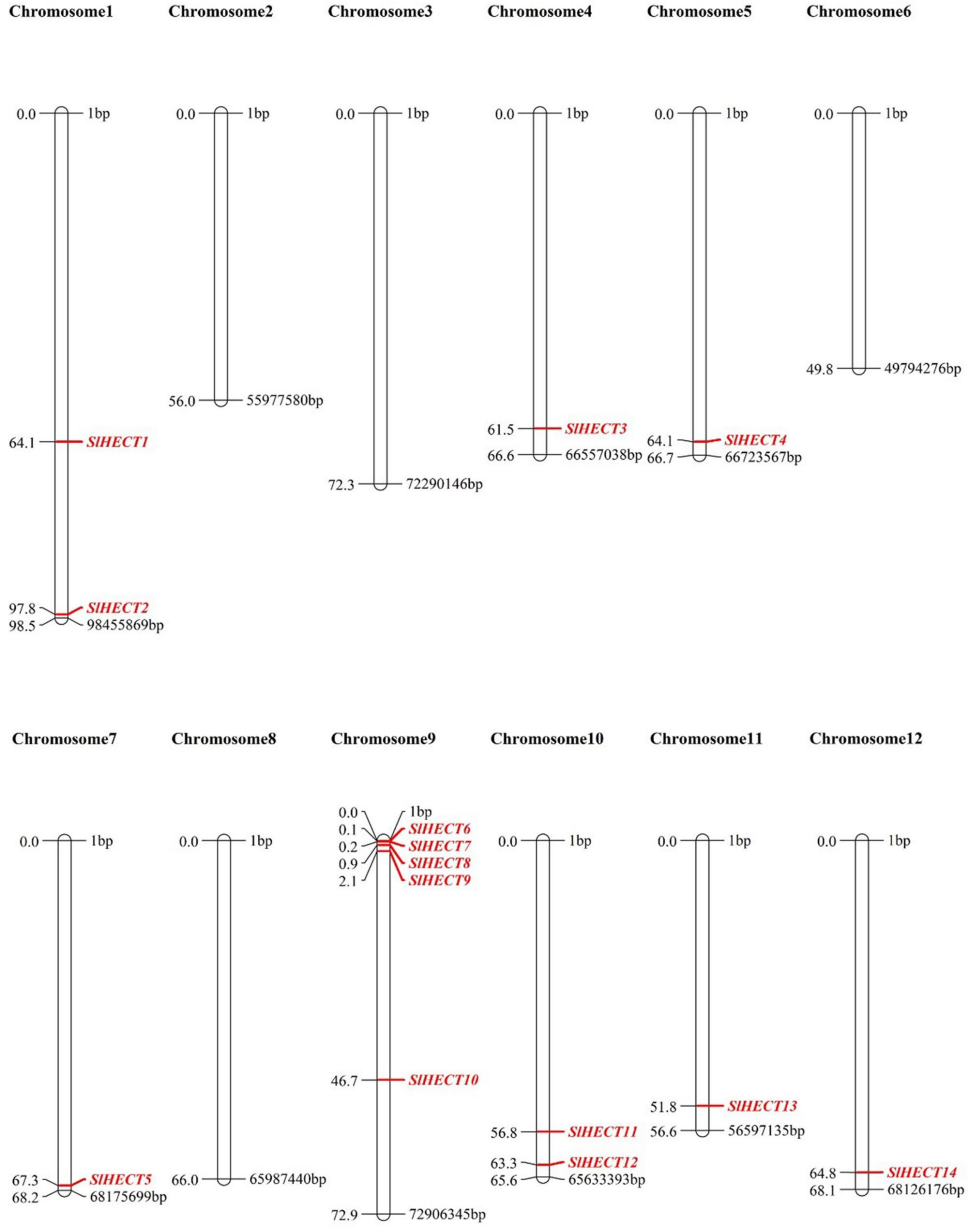
The diagram depicts the chromosomal map of tomato genome constructed using MapChart software (see “Materials and methods” section). All fourteen tomato HECT E3 ubiquitin ligases are localized on respective chromosomes.

**Figure 3:**
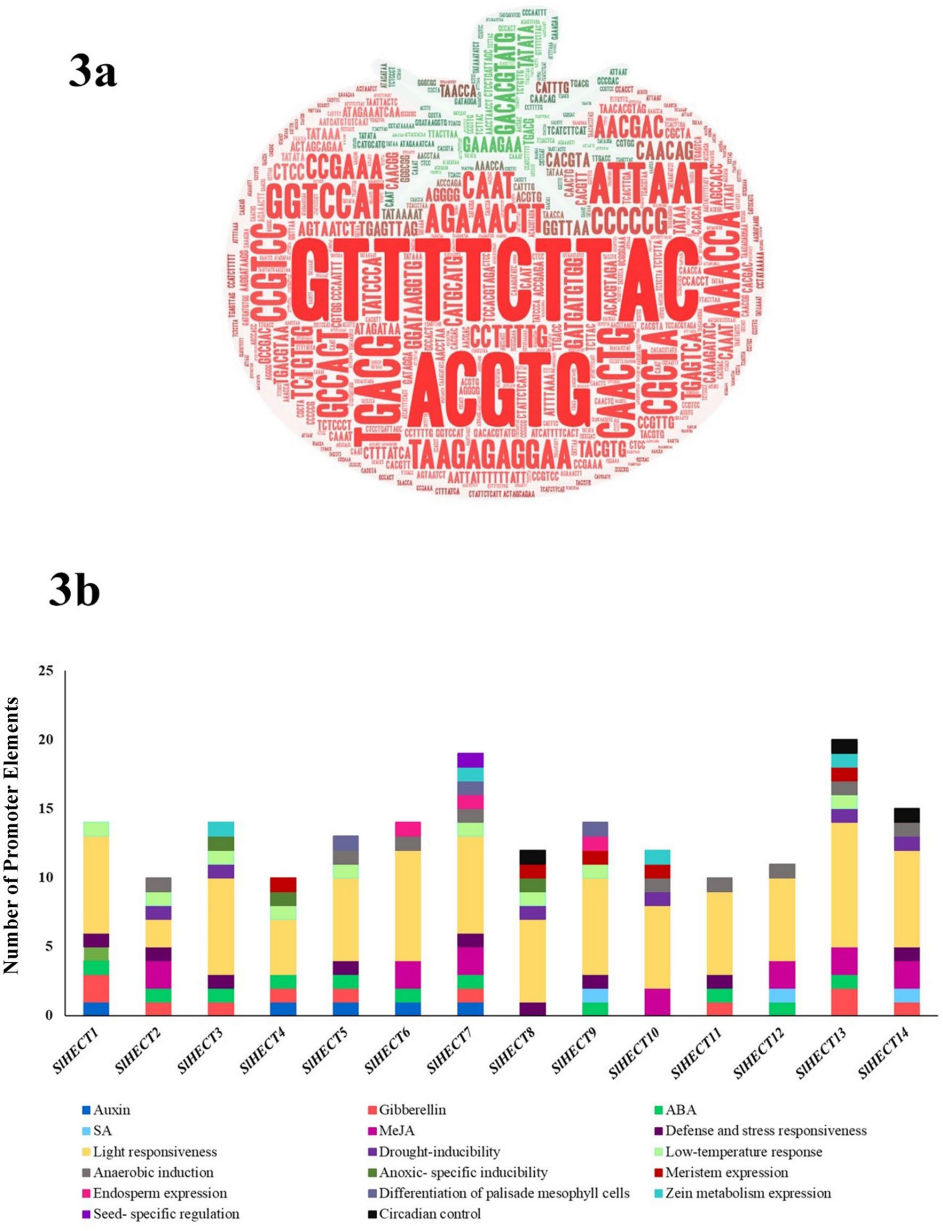
(**a**) Word cloud representing different cis-regulating elements present at 2000 bp upstream of HECT E3 ligase gene sequences (**b**) Graphical representation of fourteen HECT E3 ubiquitin ligase genes having varying roles in hormonal responses, abiotic stresses, plant development, defense, and stress responsiveness.

The HECT genes were found to be allocated across 8 out of the 12 chromosomes of *S. lycopersicum* (Fig. 2). The highest number of genes were distributed on chromosome 9 and the majority of the genes were localized at the distal ends of the chromosomes. Chromosomal recombination is a key mechanism for gene duplication and diversification of the gene pool and non-coding sequences^37^. The acquisition of the new functional domains in the HECT gene family could arise from the recombination of the distal ends of the chromosomes. The gene family expansion and functional divergence are related when genes are present at the distal ends of the chromosomes. We performed promoter region analysis for the fourteen tomato HECT genes using the PlantCARE database^38^ for the identification of cis-regulatory elements, and the transcription factor binding sites, carrying information of gene expression in response to environmental stimuli, or involvement in cellular pathways (Fig. 3a). The analysis of promoter sequences predicted the regulatory roles of the HECT gene family in tomato plant development. The different elements were found participating in hormonal pathways, plant development, abiotic stresses, defense, and stress responsiveness (Fig. 3b). All the HECT genes harbor the elements which play a major role in light responsiveness and abiotic stress. The abiotic stress factors were explicated by the elements; CAACTG (drought-inducibility), CCGAAA (low-temperature), AAACCA (low-oxygen response or anaerobic induction), and CCCCCG (no-oxygen response or anoxic-specific inducibility). Most of the HECT genes accommodate elements for hormonal responses such as auxin, gibberellins, abscisic acid, salicylic acid, and methyl jasmonate, suggesting their crucial participation in hormonal pathways (Fig. 3 and Supplementary Table S2). We found elements contributing to the development of the tomato plant, namely, GCCACT (meristem expression), TGAGTCA (endosperm expression), CAAT(A/T)ATTG (differentiation of palisade mesophyll cells), GATGATGTGG (zein metabolism regulation), CATGCATG (seed-specific regulation) and CAAAGATATC (circadian control). GTTTTCTTAC element was responsible for defense and stress responsiveness.

### Motif-based analysis and genomic organization

**Figure 4:**
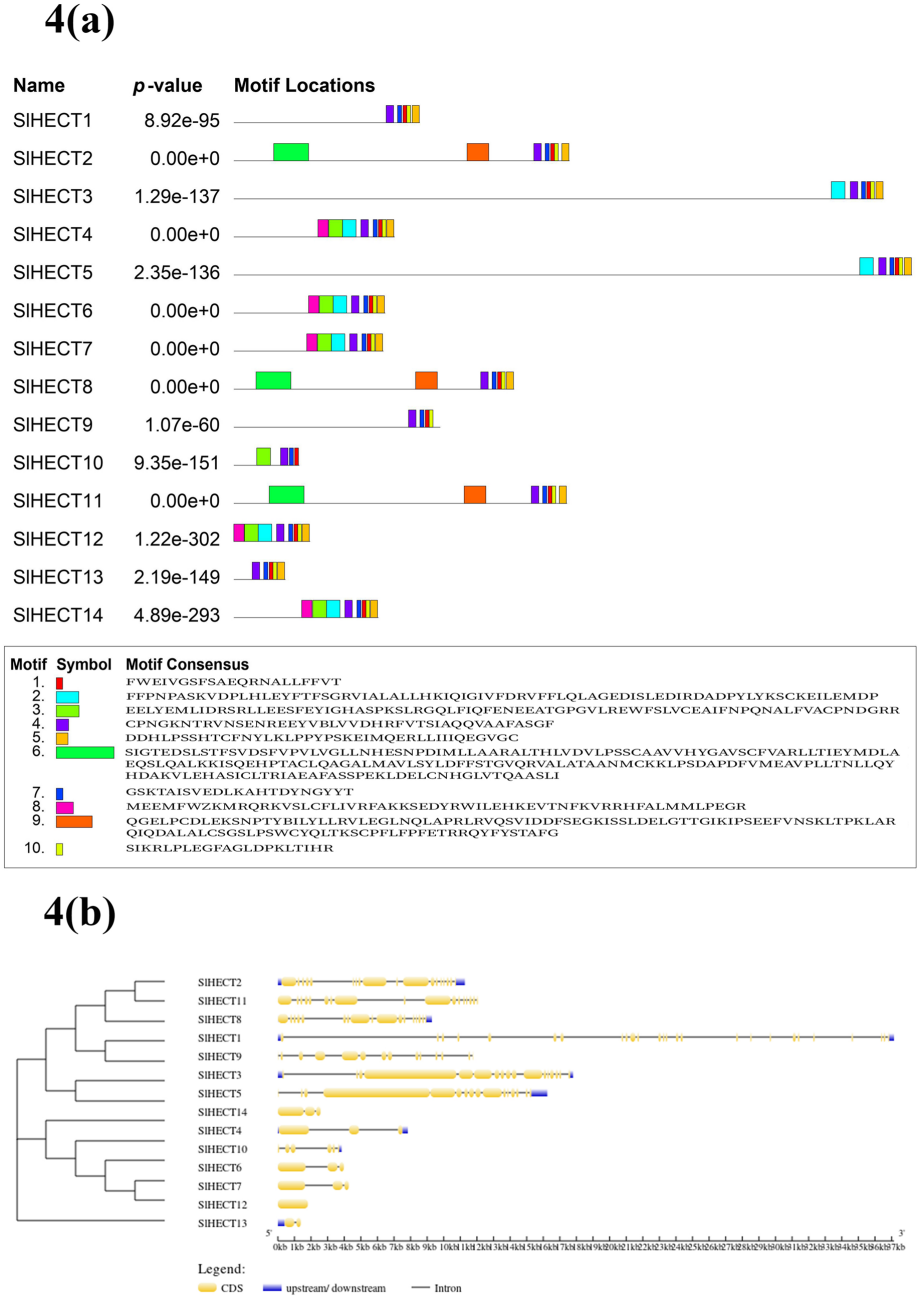
(**a**) Schematic representation of ten motifs discovered in tomato HECT E3 ubiquitin ligase gene family through MEME tool denoted by different colors (**b**) The exon/intron distribution of the fourteen tomato HECT E3 ubiquitin ligase gene family was determined by the GSDS tool, by comparing the coding sequences (CDS) with the relative genomic sequences. The yellow box represents the CDS; the solid black line depicts the intron region and the blue box shows upstream/downstream regions.

Ten novel conserved motifs were discovered using a two-component finite mixture model in MEME suite for fourteen HECT gene family members (Fig. 4a). The identified motifs were related to the HECTc superfamily and were majorly involved in the eukaryotic ubiquitin-protein ligase activity (Supplementary Table S18). The width of the discovered motifs ranged from 21 to 193 amino acids (Supplementary Table S4). Motif 1, 4, and 7 were present in all the 14 *SlHECT* sequences and may represent the core HECT domain corresponding to ubiquitin ligase activity. Interestingly, motif 10 was present in all the sequences except *SlHECT* *10,* and Motif 5 was present in all the sequences except *SlHECT* *9* and *10*. These selective presences of motifs could be responsible for functional divergence. Motif *9* and 6 appeared in exclusively *SlHECT* *2*, *8*, and *11* while motif 2 and motif 3 were present in *SlHECT* *4, 6, 7, 10, 12*, and *14* members of the tomato HECT gene family. We have observed positional and symmetrical conservation of functional domains throughout the tomato HECT gene family that evince the common biological functions. The motif enrichment analysis was performed in organisms *Arabidopsis thaliana*, *Oryza sativa*, *Populus trichocarpa, Vitis vinifera, Sorghum bicolor, Zea mays, Mus musculus*, and *Homo sapiens* (Supplementary Figure S5). The discovered motifs were further scanned in HECT protein sequences of these reference organisms to assess the evolutionary and structural relatedness (Supplementary Table S4, Supplementary Figure S5). Few discovered motifs found in tomato were also present in the HECT gene families from other species. Motif 1 and 10 were present in almost all the HECT protein sequences of plants suggesting a hallmark conservative function. Motif 5 was present in only a few protein sequences of the HECT genes. It is an interesting example of the evolutionary closeness of the different organisms. The analysis of discovered motifs in tomato predicts significant structural and functional conservation in different organisms. The results imply that the core HECT domains in E3 ligases are modified and sometimes, expanded to participate in diverse cellular and biological pathways.

We found distinct and complex exon and intron structural organization patterns in tomato HECT gene family members (Fig. 4b). The *SlHECT 12* was observed without any intron, while maximum exons were found in *SlHECT 1*. Approximately half of the *SlHECT* genes were characterized by two or fewer introns and one-third of the genes exhibited 14–18 exons. The presence of numerous exons indicates assorted functional capabilities and expansion of the HECT gene family in tomato. The arrangement of introns and exons demonstrated the conservation of the structural sequence matrix throughout the gene family.

### The architecture of conserved residues in the HECT domain and three-dimensional structure prediction

The crystal structure of human HECT Nedd4 (neural precursor cell expressed developmentally down-regulated protein 4) ^21,39^ was used as a reference for 14 *SlHECT* members (Supplementary Figure S6b). The three-dimensional HECT domain structures of all the 14 *SlHECT* were aligned with the Nedd4 (PDB ID: 4BBN) chain A to understand the structural organization of the tomato HECT domain (Supplementary Fig. 6a). A logo representing conserved regions in the HECT domains in the *S. lycopersicum* was developed to gain insights into structural relatedness among *SlHECT* members (Supplementary Figure S14). The ubiquitin transfer is mediated through the binding of the E2 ubiquitin-conjugating enzyme with the E6-AP HECT domain followed by the thiol-ester exchange to transfer the ubiquitin from E2 to cysteine residues in the E3 ubiquitin ligase enzyme^21,40^. Later, the transfer may occur to either a protein molecule or to another ubiquitin molecule to undergo polyubiquitination. The ubiquitin molecule is transferred from catalytic cysteine residue to lysine and forms an iso-peptide bond^41,42^. We observed significant conservation in the tomato HECT members when compared with Nedd4, and the N-lobe of the HECT domain comprise E2 ubiquitin-conjugating enzyme binding site. The N-terminal region is loosely conserved that facilitates interaction with a range of E2 enzymes with different specificities^43^. Most of the conserved residues were present towards the C-lobe where the critically conserved residues L282 and V291 were observed. The catalytic cysteine residues were present in the C-terminal region of the HECT domain and the amino acid alignment at the C-terminal was altered compared to the Nedd4 protein, indicating functional divergence. We observed structural modifications in the tomato HECT genes for organism-specific cellular pathways and therefore, the acquisition of specific functional characteristics. Structural characterization of the HECT proteins provides insights into the possible conformations of the proteins, their possible interaction with substrates and ubiquitin, and eventually help in determining their cellular functions^44^.

**Figure 5:**
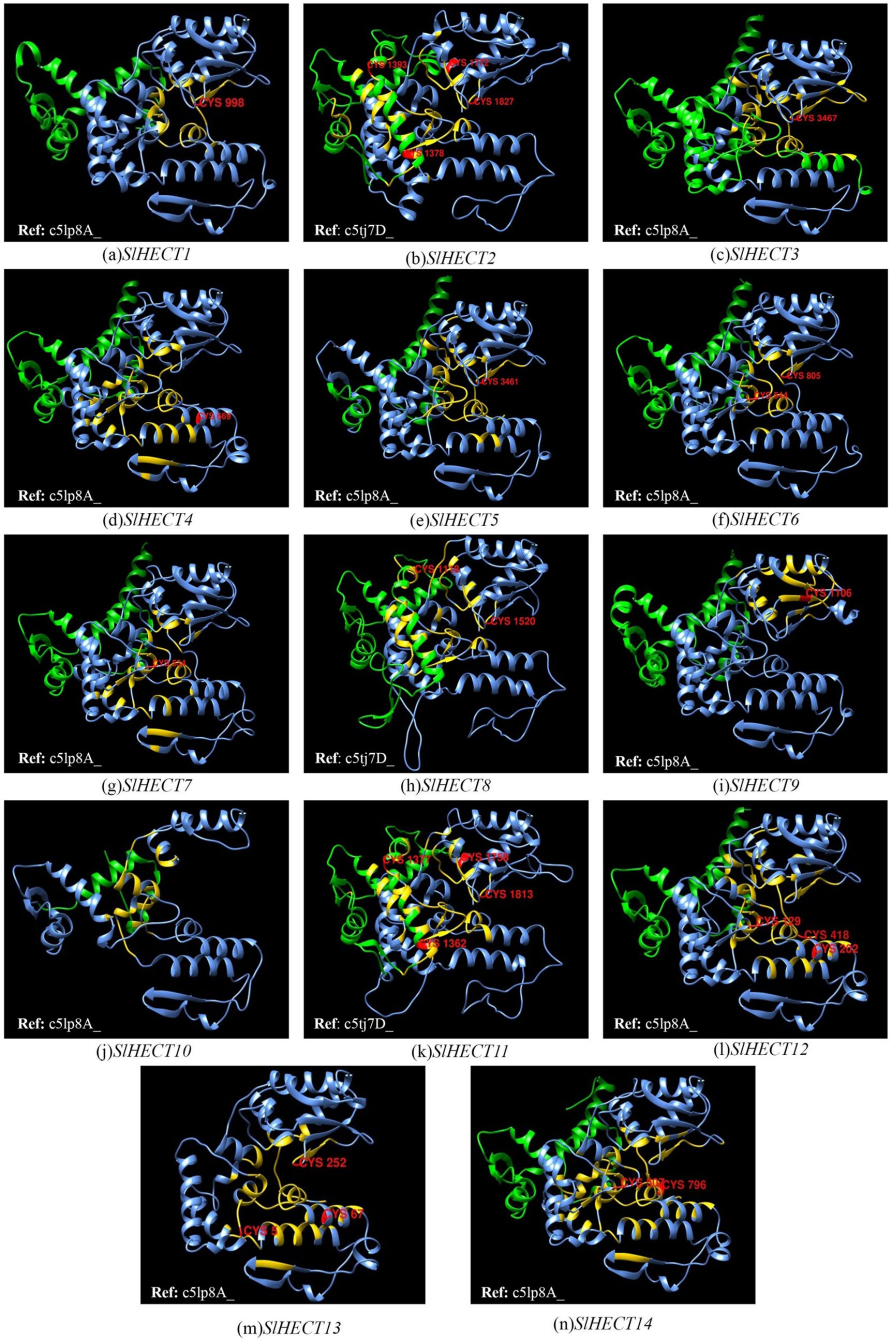
Three-dimensional structures of fourteen HECT E3 ubiquitin ligase proteins of *S. lycopersicum* (**a**–**n**)*.* The HECT domain, cysteine residues, binding pockets, and remaining residues, are highlighted with blue, red, golden, and green colors, respectively. The corresponding reference templates used for each model are highlighted in all the structures.

The knowledge of a pre-existing structure of a molecule can serve as the template for modeling the structure of the target protein based on sequence-similarity phenomena, using computational approaches^45,46^. We have performed comparative modeling of 14 *SlHECT* proteins of tomato using Phyre2, a powerful structure prediction server (Fig. 5)^45^ using the templates with a high percentage of confidence level (Supplementary Table S7). The percentage of sequence identity ranged from 27 to 54 and the characteristics of the secondary structure such as α-helix, β-strands, disordered regions, and transmembrane (TM) helix, were computed for all the predicted models. A major proportion of the secondary structure was composed of α-helices which ranged from 47 to 66%, while β-strands and disordered regions varied from 1–11% to 10–48%, respectively. Few members exhibited up to a 9% proportion of transmembrane helices. The structural variations in the HECT domains may correspond to functional diversity. The number of the exposed residues in the pockets varied among the tomato HECT gene family members depending on their structural configuration. The varied structural organizations suggest the conservation of the core ubiquitination function and participation in the diverse biological processes in tomato. All the models were validated through ERRAT and Verify3D with scores between 54.63–100, and 35.09–83.65%, respectively, which indicates the high quality of the model. The QMEAN of the structures ranged from − 10.2 to − 1.69. Ramachandran plot for all the predicted models revealed that 81.5–94.30% of the residues were in the most favorable region, while 5.40–13.70% were in the allowed region, and few residues up to 3.10% were in the disallowed region (Supplementary Table S8).

### PPI network construction, and gene ontology analysis

**Figure 6:**
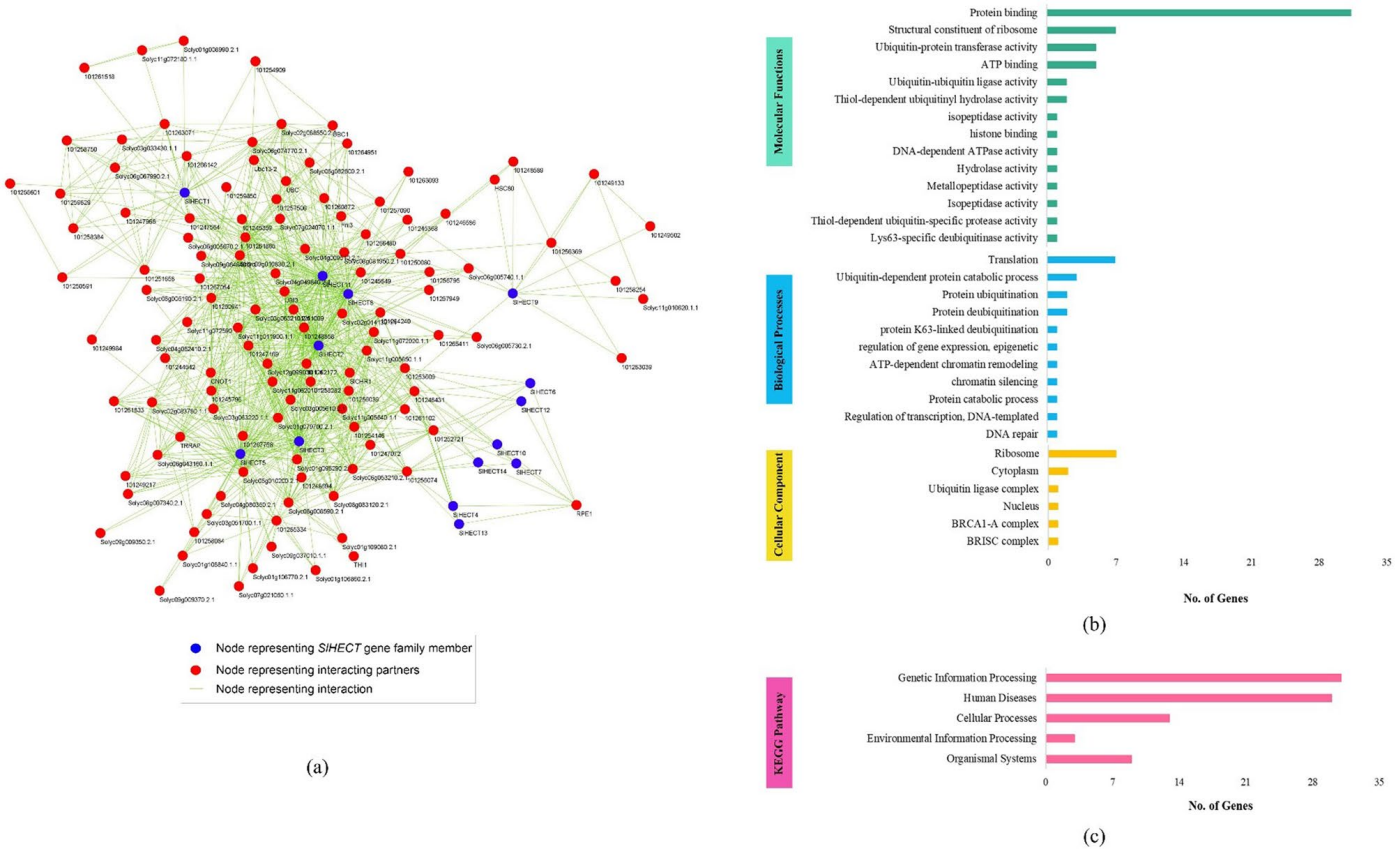
(**a**) Protein–protein interaction (PPI) network of tomato HECT E3 ubiquitin gene family. Blue nodes denote *SlHECT* members, red nodes represent interacting partners and green edges show an interaction between the nodes (**b**) gene ontology analyzed using Blast2GO suite (**c**) pathway analysis of tomato HECT E3 ligases and their interacting partners using the KAAS server.

We have designed a protein–protein interaction (PPI) network using the STRING database, with a confidence score of ≥ 0.70, and the number of interactions limited to not more than 50. The PPI network of tomato consisted of 133 nodes and 930 edges (Fig. 6a). Amongst the 133 nodes, 14 nodes are representative of the *SlHECT* gene family (blue) and the interacting partners of the respective HECT proteins are represented by red color. Large protein complexes are often regulated compactly, and the construction of meaningful modules or clusters of such protein members based on the density between the nodes and their inter-connectivity can be used to understand the crosstalk among proteins and their participation in the molecular pathways^47^. We noticed active participation and interaction of *SlHECT* *1, 2, 3, 5, 8*, and *11* gene family members with proteins involved in the various biological processes. The protein interaction map depicts the regulatory interaction of the HECT gene family in the tomato. We have used the MCODE plug-in of Cytoscape for searching locally dense regions in the PPI network and the threshold for cluster score was considered as ≥ 5. In the tomato PPI network, 3 clusters having cluster scores, 5.455, 5.200, and 5.158, respectively, were selected for gene ontology analysis (Supplementary Table S11).

The gene ontology analysis (GO) revealed that all the genes contribute to the cellular process (GO:0009987), metabolic process (GO:0008152), and biological regulation (GO:0065007) in equal proportions, as part of biological processes (Supplementary Figure S10). The involvement of the HECT E3 ligase gene family in metabolic processes further explicated their roles in nitrogen compound metabolic (GO:0006807), catabolic (GO:0009056), primary metabolic (GO:0044238), cellular metabolic (GO:0044237), and organic substance metabolic (GO:0071704) processes (Supplementary Figure S10). Molecular function analysis divulged their role in catalytic activities (GO:0003824). The HECT gene family was predicted to be localized in the cell part (GO:0044464) and cell (GO:0005623). Further, in-depth GO-based functional annotation with the total 60 nodes (protein) from all the three modules of tomato was performed using Blast2GO suite (Fig. 6b and Supplementary Table S12). The functional annotation revealed the involvement of the HECT gene family members in the ubiquitination, fundamental biological processes, and molecular mechanisms related to tomato plant development and responses to external stimuli. We noticed that most of the HECT members and interacting partners participate in the protein binding and ubiquitin transferase activity, ribosome-binding, and the translation process. Similarly, a pathway analysis, for genes present in the modules discovered through MCODE, was performed using the KEGG Automatic Annotation Server (KAAS). A total of 93 genes were analyzed and these annotated genes and pathways involved were divided into five broad groups with 45 genes in genetic information processing, 03 in environmental information processing, 15 in cellular processes, 09 in organismal systems, and 92 in human diseases (Supplementary Table S13). Most of the genes participated in pathways involved in different human diseases and genetic information processing (Fig. 6c). The tomato HECT genes and their interacting partners were least involved in the external information processing pathway. The data suggest the close correlation of the HECT domain characteristics in plants and animals that maintained the core function. The tomato HECT gene family and their interacting partners could participate in the critical cellular processes and pathways.

### Gene expression analysis in vegetative and reproductive tissues

**Figure 7:**
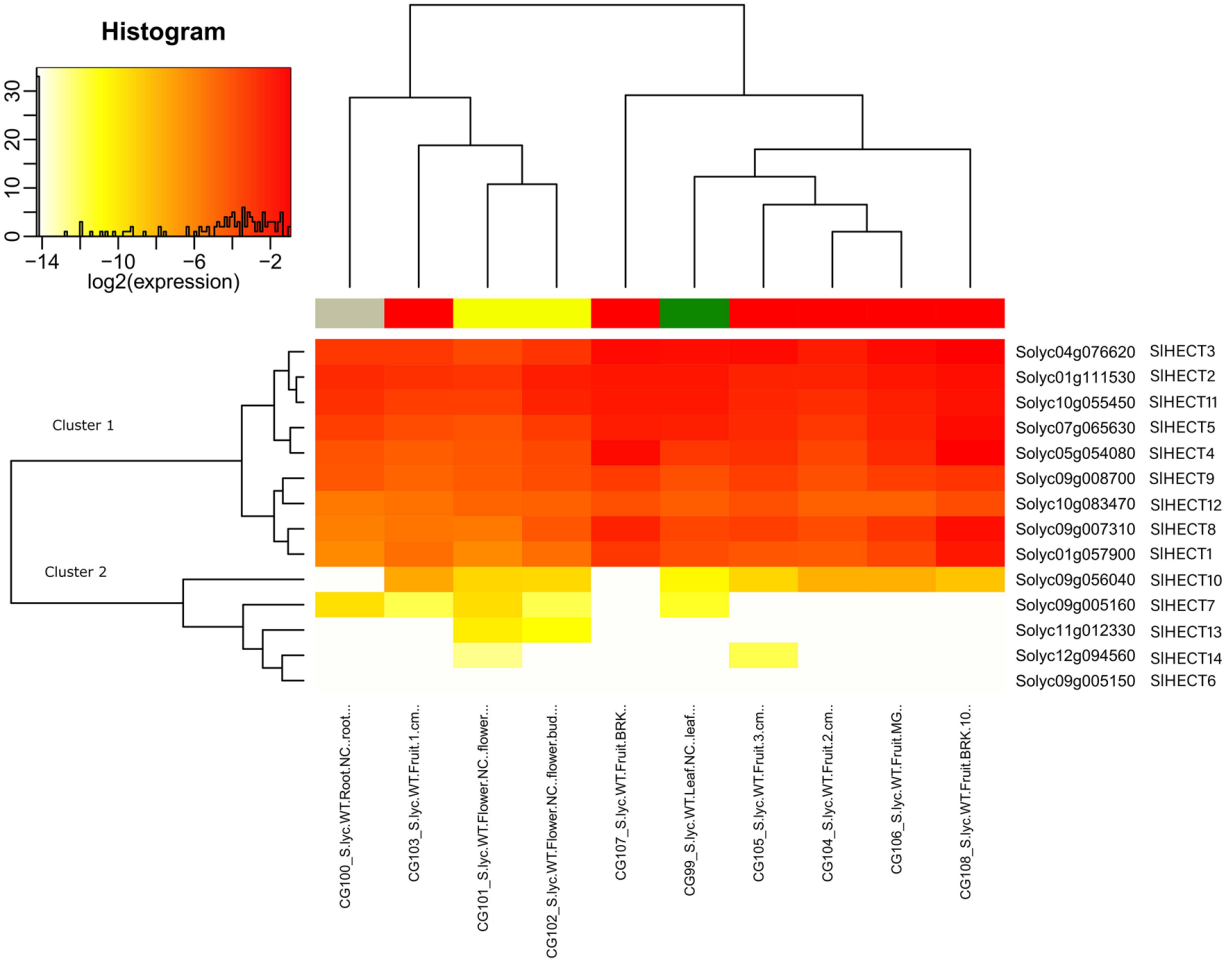
Gene expression levels of fourteen tomato HECT E3 ubiquitin ligase gene family members in vegetative and reproductive tissues (root, fruit, flower, and leaf tissues) represented by heat map (Supplementary Table S15).

The tomato HECT E3 ubiquitin ligases gene family was analyzed for the gene expression in the vegetative and reproductive tissues (Fig. 7). We noticed that Cluster 1 genes were dominantly expressed in most of the tissues, but few Cluster 1 genes were moderately expressed in the flower tissues. *SlHECT 6*, despite the presence of characteristic HECT and armadillo domains, did not express at all in most of the tissues due to either the presence of a specific functional domain that could restrict discrete participation of the sequences in any cellular activity or could be a selective expression in response to any external stimuli. However, *SlHECT 7* was exclusively expressed in leaf, flower, and root tissues and *SlHECT13* expression could be correlated to flower growth. The *SlHECT 14* was expressed lightly in only flower and mature fruit tissues. The differential expression profiles of the Cluster 2 indicate organelle-specific gene expression while Cluster 1 is quite active in the vegetative and reproductive tissues.

### Gene expression analysis in hormonal pathways, abiotic and biotic stresses

**Figure 8:**
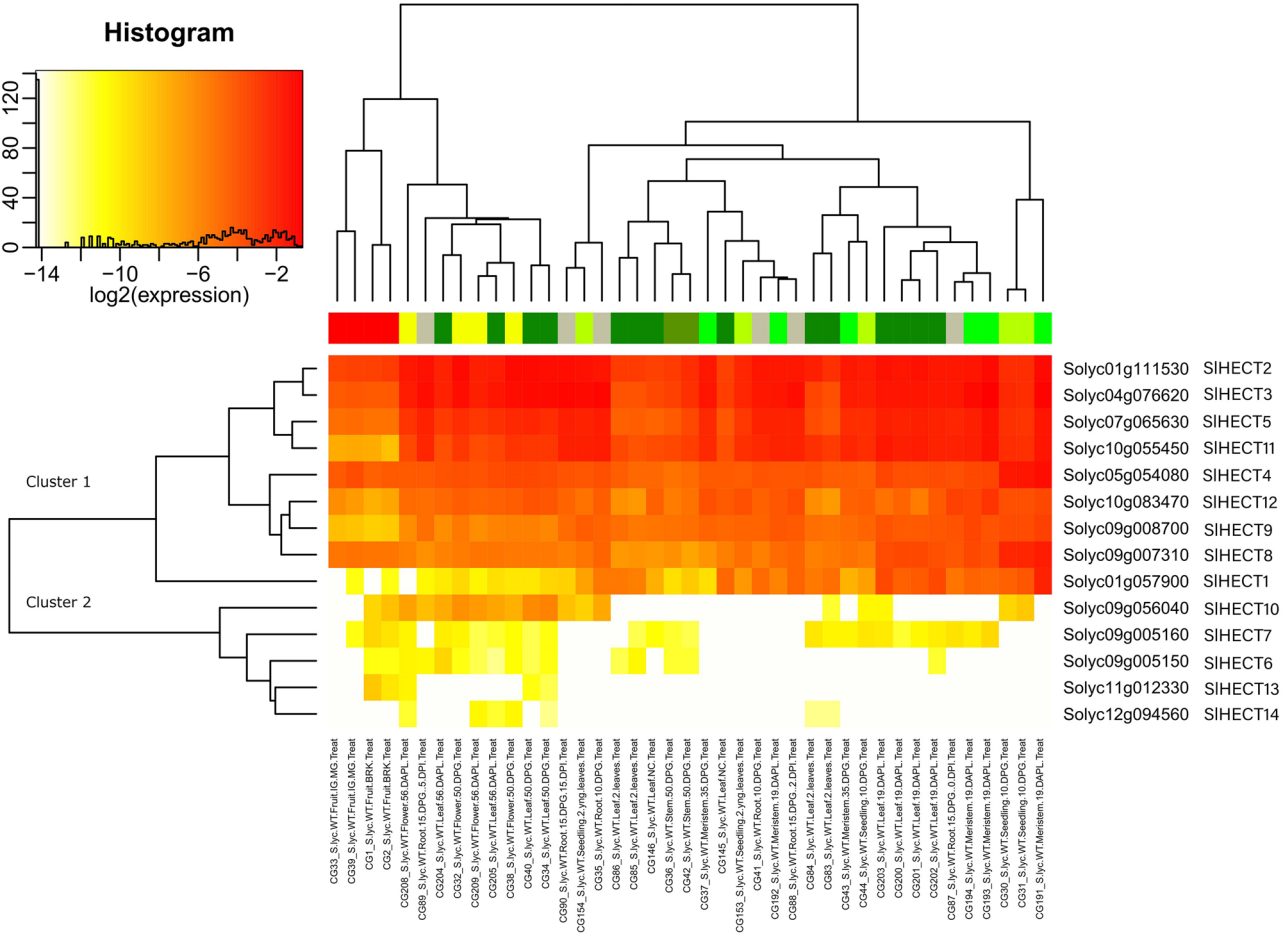
Gene expression levels of fourteen tomato HECT E3 ubiquitin ligase gene family members under abiotic and biotic stress treatments in vegetative and reproductive tissues represented by heat map (Supplementary Table S16).

The HECT E3 ubiquitin ligase gene family members were assessed for their expression under the biotic (*Meloidogyne javanica*, *Funneliformis mosseae*, Tomato Yellow Leaf Curl Virus, Virus-Induced Gene Silencing of Argonaute genes), and abiotic (sun, shade, and heat shock) stress conditions and hormonal exposure (cytokinin, auxin, indole acetic acid, 1-aminocyclopropane-1-carboxylic acid). The abiotic stress treatments significantly changed the expression profile of the HECT gene family members (Fig. 8). The Cluster 1 genes were expressed in vegetative tissues as reported earlier and selectively under-expressed in the reproductive tissues. The Cluster 2 members were mostly expressed at lower levels in reproductive tissues and leaf parts of the tomato plant. The *SlHECT* *6, 7*, and *10* were selectively expressed under abiotic stress in the reproductive tissues. Moreover, we noticed that the differential gene expression profile of the HECT gene family members was affected under biotic stress exposure (Fig. 8). The root tissues were found with lowered Cluster 1 genes expression in the 5- and 15-day infection of *Meloidogyne javanica* compared with regular gene expression levels that suggest an indication of defense alert. Further, the leaf tissues infected with Tomato Yellow Leaf Curl Virus showed a higher level of Cluster 1 gene expression while *SlHECT 8* and *10* gene expression did not change upon induction of the biotic stress (*Funneliformis mosseae*). These findings advocate the direct involvement of Cluster 1 genes in biotic stress response in tomato. Except for *SlHECT* 6 and 10, no Cluster 2 member was significantly expressed in response to biotic stress, and *SlHECT 13* was exclusively expressed in fruit tissues infected with *Funneliformis mosseae*. Cluster 2 genes were selectively expressed under biotic stress exposure and present a classical example of HECT E3 ligase gene family evolution for biotic stress response.

**Figure 9:**
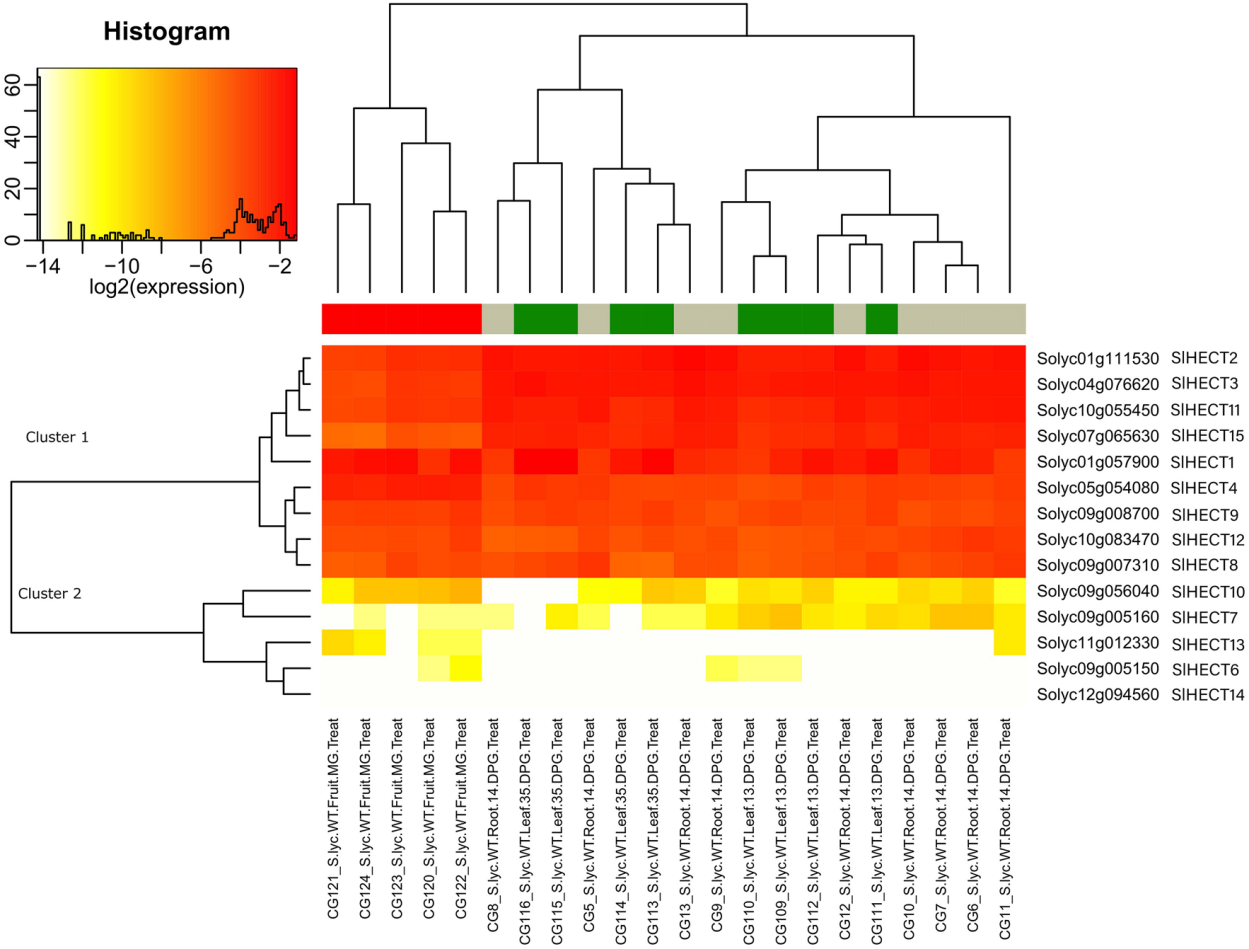
Gene expression levels of fourteen tomato HECT E3 ubiquitin ligase gene family members under hormonal treatments in vegetative and reproductive tissues represented by heat map (Supplementary Table S17).

We observed that Cluster 1 genes were expressed in all the tissues under all the hormonal treatments suggesting activation of the HECT E3 ligases in response to hormonal treatments (Fig. 9). The genes with partial HECT domain were not expressed under hormone exposure and *SlHECT 6* and *13* were selectively expressed in root and fruit tissues. The fruit tissues treated with IAA and ACC hormones showed mild gene expression and the *SlHECT 5* was under-expressed compared to other members in the fruit tissues. However, *SlHECT 7* and *10* showed a comparatively lower expression profile and expressed in almost all the tissues. We did not find selective changes in the Cluster 1 genes for the cytokinin and auxin hormones treatments. Our results convey a very strong indication of Cluster 1 gene participation in stress and hormone treatments.

## Discussion

The E3 ubiquitin ligases are the largest and crucial members of the ubiquitin-proteasomal degradation mechanism. The HECT E3 gene family has been identified and studied amongst some higher plants, such as *Arabidopsis thaliana, Brassica rapa, Brassica oleracea, Glycine max, Zea mays, Physcomitrella patens, Oryza sativa, Solanum tuberosum,* and *Malus domestica*^17,18,22,24–26^*.* There are no evident traces of the characterization of the HECT gene family in tomato, ever since its genome was completely annotated in 2012, therefore, we have aimed to identify and delineate the HECT E3 ligases present in *S. lycopersicum*^48^*.* A total of fourteen genes with core HECT domain were identified in tomato by employing *in-silico* analyses. The physical and chemical characteristics revealed the stable and hydrophilic nature of the tomato HECT E3 ligases. Most of the *SlHECT* gene family members were found in the nucleus, cytoplasm, and few members were localized in the chloroplast. Previous studies have reported the colocalization of HECT E3 ligases in both the nucleus and cytoplasm. For instance, the mouse Nedd4 comprises certain amino acid sequences ranging from 402 to 413 which directs its localization into the nucleus^49^. In addition to this, human Nedd4 related WWP1 protein shows context-dependent translocation in the nucleus when co-expressed with human Notch1, which implies that the co-expression of proteins can change the localization patterns^50^. The phylogenetic analysis revealed a close evolutionary relation of tomato HECT members with other plant HECT gene family members. Our analysis provides interesting insights into the conservation and divergence of the HECT gene families in plants and animals. The conservation of core HECT domain and motifs in plants, the addition of new motifs, and the expansion of gene family could be a result of sub-functionalization. These evolutionary mechanisms partition the ancestral genes while retaining the duplicated genes^51^. The evolutionary factors could lead to alternative mechanisms for the partitioning of ancestral functions. However, we could not observe any duplication or syntenic event in the tomato HECT gene family.

The HECT gene family classification was based on the different domains present at the N-terminal of the HECT E3 ligases^35^. NEDD4 sub-family (Class I), comprises domains WW and C2 mainly, which helps in the identification of substrates for degradation. The HERC sub-family (Class II), comprising SPRY, APC10, Cyt-b5, ZZ, and WD40 domains, contributes to ubiquitin-mediated protein degradation. Further, the classes were formed based on the presence of the HECT domain and associated domains (Class III-VI), which are abundant with a repertoire of domains such as Ubiquitin-Associated (UBA), Ubiquitin Interacting Motif (UIM), Ubiquitin (UBQ), Armadillo-repeat domain (ARM) and IQ domains, that help in ubiquitin ligase activity^28,36,52^. ARM, IQ, UBA, and UIM domains have been discovered in the *Arabidopsis thaliana* HECT gene family suggesting a close functional association^27^. The phylogenetic analysis of tomato HECT members construed distinct responsibility in recognition of substrates for protein degradation, or binding to ubiquitin moieties to form polyubiquitin chains. On the contrary, most members of the mouse and humans HECT E3 gene family were present in classes I and II, suggesting their major roles in human diseases, apart from ubiquitin ligase activity. Further, to ascertain the molecular and biological functions associated with the HECT gene family members, we constructed a protein–protein interaction network^53^. Our results indicate that the HECT gene family influences ATP binding, isopeptidase activity, histone binding, protease activity, and numerous molecular and biological processes in tomato. The HECT E3 ubiquitin ligases have been identified to regulate trichome development^54^, leaf senescence^55^, biotic stress^56^, cell growth, proliferation, autophagy, DNA repair, antiviral responses, and many diseases^40^. Our results agree with previous studies and predict extended involvement in the tomato cellular and genetic processes.

The promotor analysis of the tomato HECT gene family revealed a range of cis-regulatory elements participating in various developmental and environmental response mechanisms. The analysis of promoter sequences unveiled the role of *SlHECT* ligases in defense and stress mechanisms, abiotic stress responses, plant development, and hormonal regulation. Each member showed responsiveness towards light indicating the HECT E3 ligase gene family contribution to light signaling and phytohormone pathways. The previous reports on E3 ligases in plants have suggested their roles in hormonal signaling and plant development, along with responses towards abiotic stress factors^14,15^. The findings were further validated with protein–protein interactions, gene ontology studies, and gene expression analysis. The gene ontology data implicated that tomato HECT E3 ligases are involved in biological regulation and various cellular and metabolic processes, indicating the presence of various interaction motifs upstream to the HECT domain, which has roles in ubiquitin-binding, regulation, and localization^22^. All the *SlHECT* members displayed the catalytic activity, a key feature of the HECT domain as a part of molecular function, that catalyzes both Ub-substrate and Ub-Ub binding processes^12^. Additionally, we visualized the location of *SlHECT* members on tomato chromosomes wherein chromosome 9 was found to be densely populated with the presence of the majority of *SlHECT* genes, and genes were located on the distal regions. The distal region genes are prone to genetic recombination and functional diversification^57^. We could not find any duplication among tomato HECT gene family members. Motifs are functionally important, short stretches of DNA, RNA, or protein sequences, which are widespread and inferred to have the same biological function, serving as key elements of molecular evolution^58^. The presence of certain motifs in all the members of tomato implies their conserved nature by evolution, while the sparse distribution of some motifs across the members indicates specialized functions in the plant cellular mechanisms. The exon–intron arrangement has been known to contribute to structural divergence, especially in duplicated genes^59^. The pattern of exon–intron on the HECT gene family members exhibits a complex organization that suggests the presence of the diverse functional motifs responsible for their involvement in different molecular mechanisms.

The sequence-structure–function relationships unravel the presumptive interactions with ligands and other proteins^44^. The insights into the architecture of 14 *SlHECT* members showed conserved HECT domain at the C-terminal, diverse alpha-helix, and disordered region. The protein structural differences influence their target binding capabilities and are directly related to their three-dimensional structures^60^. The GO-driven functional annotation predicted three predominant molecular functions; protein binding, ubiquitin-protein transferase activity, and ubiquitin-ubiquitin ligase activity, in which all the members actively participate. Interestingly, *S. lycopersicum* HECT gene family exclusively participates in molecular functions like DNA-dependent ATPase activity, histone binding, and isopeptidase activity^61–63^. Formerly, studies on ubiquitin-mediated protein degradation have reported the alternative roles of ubiquitin machinery in DNA repair, and signal transduction^35^. The GO category of cellular components has shed light on the translocation of all the HECT E3s and their interacting proteins in the ubiquitin ligase complex, indicating their roles in the ubiquitin-ligase activity. Conversely, members of tomato PPI modules have shown localization, majorly in ribosomes, in addition to cytoplasm and nucleus. Similarly, KEGG pathway analysis revealed that most of the proteins in the PPI networks participated in the genetic information processing pathways, mainly ubiquitin-mediated proteolysis, and proteasomal degradation.

We have used the TomExpress RNA sequencing database for the extraction of the differential gene expression profiles for the tomato HECT gene family under environmental stress and plant developmental conditions^64^. We observed a strong constitutive expression profile of the Cluster 1 genes indicating their active role in regulating protein quality and participating in plant development, and stress responses. The HECT genes have been found to regulate seed size and crop yield in the *Brassica napus* and cell death in *Brassica rapa*^65^. A similar constitutive expression profile of the HECT was observed in the *Brassica oleracea* that is responsible for cellular pathways^26^. Few genes from Cluster 2 were selectively expressed under abiotic and biotic stress conditions whereas the Cluster 1 genes were actively expressed in all the tissues suggesting the critical requirement of the HECT gene family members for generating responses against external stimuli. The HECT gene family was found to participate in abiotic stresses such as cold and drought in *Malus domestica*^25^. Our results agree with previous studies on the HECT gene family members in plant species. The promoter and gene ontology analysis validated the gene expression analysis that confirms the involvement of the HECT gene family members in the plant growth and development, and stress responses. The presence of the diverse motifs or functional domain in the tomato HECT gene family may qualify them to participate in numerous molecular mechanisms.

Identification and characterization of the HECT gene family members aid in exploring the genetic expansion, distribution, and involvement in tomato plant development. We speculate a strong relation of the HECT gene family in the growth and development of the tomato plant. The HECT gene family can be targeted for the elucidation of several molecular mechanisms related to development, plant immunity, adaptation, drought, and salinity stress responses. This work will serve as preliminary evidence for future studies of the E3 ubiquitin ligases in plants.

## Materials and methods

### Identification and characterization of HECT gene family in *Solanum lycopersicum*

The candidate proteins of the HECT family of E3 ubiquitin ligases were extracted using the HMMER program, downloaded from HMMER (http://hmmer.org/)^66^. The HECT domains were identified with the help of the Pfam database (https://pfam.xfam.org/), to generate the HMM profile of putative candidates^67^. With *S. lycopersicum* as a reference database (iTAG2.4), an HMM search was performed with default parameters and a significant e-value of 0.01. The presence of the HECT domain in the candidate proteins was validated using a Simple Modular Architecture Research Tool (SMART) (http://smart.embl-heidelberg.de/)^68^. The physicochemical parameters, such as molecular weight, number of amino acids, instability index, aliphatic index, and grand average of hydropathicity (GRAVY), of the HECT proteins, were computed with the aid of ExPASy ProtParam (https://web.expasy.org/protparam/) tool^69^. To determine the chromosomal location as well as intron–exon count, PhytoMine (https://phytozome.jgi.doe.gov/phytomine/begin.do), an InterMine surface from Phytozome, was used^70^.

### Subcellular localization and gene ontology analysis

Balanced subCellular Localization predictor (BaCelLo) (http://gpcr.biocomp.unibo.it/bacello/) and Protein Subcellular Localization Prediction System, LocTree3 (https://rostlab.org/services/loctree3/) were used to determine the subcellular localization of the candidate proteins^71,72^. The Gene Ontology (GO) of the target proteins was identified using the server, Protein ANalysis Through Evolutionary Relationships (PANTHER) (http://www.pantherdb.org/)^73^.

### Chromosomal localization and analysis of promoter sequences

The chromosomal lengths of the 12 chromosomes of tomato were retrieved from Ensembl Plants database (https://plants.ensembl.org/index.html)^74^. The chromosomal positions were retrieved from the PhytoMine server and were used for determining the positions of the candidate proteins on the chromosomes of *S. lycopersicum,* using MapChart 2.32 software, downloaded from MapChart (https://www.wur.nl/en/show/Mapchart.htm)^70,75^. The promoter sequences (2000 bp in size) for the HECT gene family of tomato were extracted from Phytozome (https://phytozome.jgi.doe.gov/pz/portal.html)^70^. These promoter sequences were represented in the form of a word cloud, with the help of the WordArt tool (https://wordart.com), and were analyzed for the presence of different motifs, using the PlantCARE database (http://bioinformatics.psb.ugent.be/webtools/plantcare/html/)^38,76^.

### Motif and gene structure analysis

Novel conserved motifs were discovered by using probabilistic and discrete algorithms of MEME suite (http://meme-suite.org/tools/meme), a motif-based sequence analysis tool^58,77^. Maximum optimal width (number of characters in a sequence pattern) of 200 amino acids was used for a single motif search with 10 number of motifs limit. The identified motifs were analyzed for their conserved roles in the eukaryotic system using the Conserved Domains Database (CDD) (https://www.ncbi.nlm.nih.gov/Structure/cdd/wrpsb.cgi) and EggNOG mapper, a rapid functional annotation tool for gene and protein sequences, found within OmicsBox 1.3.11 (https://www.biobam.com/omicsbox/), using the default parameters^78–80^. Motif alignment and search tool (MAST) of MEME suite (http://meme-suite.org/tools/mast) were used to search for the occurrence of tomato motifs in the HECT gene family of *Arabidopsis thaliana*, *Oryza sativa*, *Populus trichocarpa, Vitis vinifera, Sorghum bicolor, Zea mays, Mus musculus*, and *Homo sapiens*.

The HECT gene family members for each of the reference organisms were retrieved from the Ubiquitin and Ubiquitin-like Conjugation Database, UUCD (http://uucd.biocuckoo.org/index.php)^81^. Motif enrichment analysis was done using Analysis of Motif Enrichment (AME) (http://meme-suite.org/tools/ame) to find relative enrichment of discovered motifs in the reference organisms^82^. Furthermore, the characterization and visualization of HECT gene structure and annotated features like CDS and introns’ length was performed using Gene Structure Display Server (GSDS) v2.0 (http://gsds.cbi.pku.edu.cn/)^83^.

### Multiple sequence alignment and identification of conserved residues in the HECT domain

To assess the alignment of the retrieved HECT domains from the putative candidates of tomato, multiple sequence alignment (MSA) was performed in the software, Jalview 2.11.0, downloaded from Jalview (http://www.jalview.org/), by taking human HECT Nedd4 (neural precursor cell expressed developmentally down-regulated protein 4) as a reference, whose sequence information (PDB ID: 4BBN, chain A) was retrieved from RCSB- Protein Data Bank (PDB) (https://www.rcsb.org/)^84,85^. The sequence alignment was visualized as logo using the tool, WebLogo 3 (http://weblogo.threeplusone.com/) and as a three-dimensional structure, using UCSF Chimera 1.14 software (https://www.cgl.ucsf.edu/chimera/)^86–88^.

### Protein structure prediction for structural characterization

The peptide sequences of the putative targets were submitted in a web-based server, Protein Homology/analogy Recognition Engine (Phyre2) version 2.0 (http://www.sbg.bio.ic.ac.uk/~phyre2/html/page.cgi?id=index) for predicting the structural attributes and models of the HECT gene family in tomato^45^. UCSF Chimera 1.14 software (https://www.cgl.ucsf.edu/chimera/) was used for visualization and generation of graphic images of the models predicted^88^. The qualitative assessment of the predicted models was performed using the servers, the Structure Analysis and Verification Server (SAVES) v6.0 (https://saves.mbi.ucla.edu/) and Qualitative Model Energy ANalysis (QMEAN) (https://swissmodel.expasy.org/qmean/)^89–92^. SAVES v6.0 provides the platform for stereochemical assessment through PROCHECK, analysis of the 3-D structure using atomic-resolution coordinates through VERIFY-3D, and provides the overall quality factor of non-bonded atoms in structure using ERRAT^89–91^. QMEAN evaluates the quality of the model by assessing the likelihood of the generated model being comparable to the experimental structure through the ‘degree of nativeness’ and the quality score of the model is then expressed in terms of 'Z-scores'^92^.

### Phylogenetic tree construction

The evolutionary relationship was assessed using the software, Molecular Evolutionary Genetic Analysis (MEGA) (https://www.megasoftware.net/)^93^. Multiple sequence alignment was performed by ClustalW using all the default parameters in MEGA X and used to construct a phylogenetic tree using the Maximum Likelihood method with 1000 bootstraps per replication. Interactive Tree Of Life (iTOL) v6.3 (https://itol.embl.de/) was used for visualization of the phylogenetic tree^94^.

### Tissue-specific expression patterns in HECT gene family

Gene expression profile of the tomato HECT ubiquitin ligase was inquired using the latest RNA sequencing data pipeline of the TomExpress database (http://tomexpress.toulouse.inra.fr/)^64^. The gene expression was analyzed in vegetative, reproductive tissues, biotic (*Meloidogyne javanica*, *Funneliformis mosseae*, Tomato Yellow Leaf Curl Virus, Virus-Induced Gene Silencing of Argonaute genes) and abiotic (sun, shade, and heat shock) stress conditions and hormonal exposure (cytokinin, auxin, indole acetic acid, 1-aminocyclopropane-1-carboxylic acid). The data was visualized using heat maps generated from TomExpress database.

### Protein–protein interaction (PPI) network construction and cluster analysis

For the assessment of the functional association between the HECT gene family as well as with other related proteins, protein–protein interaction (PPI) networks were constructed using the STRING v11 (Search Tool for the Retrieval of Interacting Genes) database (https://string-db.org/)^95^. The peptide sequences of target proteins of tomato were used as input and the maximum first shell interactors for each protein sequence were selected to be no more than 50’ via basic settings in the STRING interface. The confidence cut-off score for each interaction was set to ‘high’ (0.700) along with other default parameters. The interaction network data were used for visualization and cluster analysis through Cytoscape (https://cytoscape.org/)^96^. Cytoscape plug-in, Molecular COmplex DEtection (MCODE) was utilized to identify meaningful modules such as clusters, bearing potential functions within the PPI networks. In this, the value of k-core and node score cut-off were 2 and 0.2, respectively, along with other default settings. Modules having a cluster score ≥ 5 were selected for further analysis.

### Functional annotation and pathway analysis of PPI network

Blast2GO, a functional analysis module of OmicsBox 1.3.11 (https://www.biobam.com/omicsbox/), was used for performing Gene Ontology-based functional annotation with the nodes (protein) in each module^97^. Peptide sequences (in FASTA format) of all the interacting proteins in each cluster were retrieved from UniProt (https://www.uniprot.org/) and run in Blast2GO, using the default parameters^98^. For a deep understanding of the roles played by these proteins in biological systems, pathway analysis was performed in a Kyoto Encyclopedia of Genes and Genomes (KEGG) web-based service, KEGG Automatic Annotation Server (KAAS) (https://www.genome.jp/kegg/kaas/), and pathways were reconstructed by BLAST comparisons in the KEGG database^99^.

## Supplementary Information

Supplementary Information.

## Data Availability

All data generated or analyzed during this study are included in this published article (and its Supplementary Information files).

## References

[1] Craig A, Ewan R, Mesmar J, Gudipati V, Sadanandom A (2009). E3 ubiquitin ligases and plant innate immunity. J. Exp. Bot..

[2] Reece JB (2011). Campbell Biology.

[3] Nelson DL (2008). Lehninger Principles of Biochemistry.

[4] Voet D, Voet JG (2010). Biochemistry.

[5] Ciechanover A (2005). Proteolysis: From the lysosome to ubiquitin and the proteasome. Nat. Rev. Mol. Cell Biol..

[6] Haglund K, Dikic I (2005). Ubiquitylation and cell signaling. EMBO J..

[7] Zheng N, Shabek N (2017). Ubiquitin ligases: Structure, function, and regulation. Annu. Rev. Biochem..

[8] Vierstra RD (2009). The ubiquitin-26S proteasome system at the nexus of plant biology. Nat. Rev. Mol. Cell Biol..

[9] Li X, Hasegawa Y, Lu Y, Sato T (2017). Ubiquitin related enzymes and plant-specific ubiquitin ligase ATL family in tomato plants. Plant. Biotechnol. (Tokyo).

[10] Moon J, Parry G, Estelle M (2004). The ubiquitin-proteasome pathway and plant development. Plant Cell.

[11] Stone SL (2014). The role of ubiquitin and the 26S proteasome in plant abiotic stress signaling. Front Plant Sci.

[12] Pickart CM (2001). Mechanisms underlying ubiquitination. Annu. Rev. Biochem..

[13] Callis J (2014). The ubiquitination machinery of the ubiquitin system. Arabidopsis Book.

[14] Mazzucotelli E (2006). The E3 ubiquitin ligase gene family in plants: Regulation by degradation. Curr. Genomics.

[15] Shu K, Yang W (2017). E3 ubiquitin ligases: Ubiquitous actors in plant development and abiotic stress responses. Plant Cell Physiol..

[16] Chen L, Hellmann H (2013). Plant E3 ligases: Flexible enzymes in a sessile world. Mol. Plant.

[17] Marin I (2013). Evolution of plant HECT ubiquitin ligases. PLoS ONE.

[18] Meng X (2015). Genome-wide identification and evolution of HECT genes in soybean. Int. J. Mol. Sci..

[19] Sharma B, Taganna J (2020). Genome-wide analysis of the U-box E3 ubiquitin ligase enzyme gene family in tomato. Sci. Rep..

[20] Rotin D, Kumar S (2009). Physiological functions of the HECT family of ubiquitin ligases. Nat. Rev. Mol. Cell Biol..

[21] Maspero E (2011). Structure of the HECT:ubiquitin complex and its role in ubiquitin chain elongation. EMBO Rep..

[22] Downes BP, Stupar RM, Gingerich DJ, Vierstra RD (2003). The HECT ubiquitin-protein ligase (UPL) family in Arabidopsis: UPL3 has a specific role in trichome development. Plant J..

[23] Huibregtse JM, Scheffner M, Beaudenon S, Howley PM (1995). A family of proteins structurally and functionally related to the E6-AP ubiquitin-protein ligase. Proc. Natl. Acad. Sci. U.S.A..

[24] Li Y (2019). Genome-wide identification, phylogenetic and expression analysis of the maize HECT E3 ubiquitin ligase genes. Genetica.

[25] Xu J, Xing S, Cui H, Chen X, Wang X (2016). Genome-wide identification and characterization of the apple (Malus domestica) HECT ubiquitin-protein ligase family and expression analysis of their responsiveness to abiotic stresses. Mol. Genet. Genomics.

[26] Alam I, Cui DL, Batool K, Yang YQ, Lu YH (2019). Comprehensive genomic survey, characterization and expression analysis of the HECT gene family in Brassica rapa L. and Brassica oleracea L.. Genes (Basel).

[27] Ying M (2018). Role of HECT ubiquitin protein ligases in Arabidopsis thaliana. J. Plant Sci. Phytopathol..

[28] Sluimer J, Distel B (2018). Regulating the human HECT E3 ligases. Cell Mol. Life Sci..

[29] Shikata M, Ezura H (2016). Micro-tom tomato as an alternative plant model system: Mutant collection and efficient transformation. Methods Mol. Biol..

[30] Yang L (2019). Genome-wide identification, evolution, and expression analysis of RING finger gene family in solanum lycopersicum. Int J. Mol. Sci..

[31] Krishna R (2019). Transgenic tomatoes for abiotic stress tolerance: Status and way ahead. 3 Biotech.

[32] Wai, A. H., Naing, A. H., Lee, D.-J., Kim, C. K. & Chung, M.-Y. Molecular genetic approaches for enhancing stress tolerance and fruit quality of tomato. *Plant Biotechnol. Rep.*, 1–23 (2020).

[33] Biondi A, Guedes RNC, Wan F-H, Desneux N (2018). Ecology, worldwide spread, and management of the invasive South American tomato pinworm, Tuta absoluta: Past, present, and future. Annu. Rev. Entomol..

[34] Finn RD, Clements J, Eddy SR (2011). HMMER web server: Interactive sequence similarity searching. Nucleic Acids Res..

[35] Weber J, Polo S, Maspero E (2019). HECT E3 ligases: A tale with multiple facets. Front Physiol..

[36] Grau-Bove X, Sebe-Pedros A, Ruiz-Trillo I (2013). A genomic survey of HECT ubiquitin ligases in eukaryotes reveals independent expansions of the HECT system in several lineages. Genome Biol. Evol..

[37] Wilcox J (1988). Performance and use of seedcoat mutants in soybean. Crop Sci..

[38] Lescot M (2002). PlantCARE, a database of plant cis-acting regulatory elements and a portal to tools for in silico analysis of promoter sequences. Nucleic Acids Res..

[39] Maspero E (2013). Structure of a ubiquitin-loaded HECT ligase reveals the molecular basis for catalytic priming. Nat. Struct. Mol. Biol..

[40] Wang, Y., Argiles-Castillo, D., Kane, E. I., Zhou, A. & Spratt, D. E. HECT E3 ubiquitin ligases–emerging insights into their biological roles and disease relevance. *J. Cell Sci.* **133** (2020). 10.1242/jcs.228072 PMC7157599 32265230

[41] Schwarz SE, Rosa JL, Scheffner M (1998). Characterization of human hect domain family members and their interaction with UbcH5 and UbcH7. J. Biol. Chem..

[42] Wang G, Yang J, Huibregtse JM (1999). Functional domains of the Rsp5 ubiquitin-protein ligase. Mol. Cell. Biol..

[43] Jentsch S (1992). The ubiquitin-conjugation system. Annu. Rev. Genet..

[44] Verma JK, Wardhan V, Singh D, Chakraborty S, Chakraborty N (2018). Genome-wide identification of the alba gene family in plants and stress-responsive expression of the rice alba genes. Genes (Basel).

[45] Kelley LA, Mezulis S, Yates CM, Wass MN, Sternberg MJ (2015). The Phyre2 web portal for protein modeling, prediction and analysis. Nat. Protoc..

[46] Xiong, J. *Essential Bioinformatics*. (2006).

[47] Bader GD, Hogue CW (2003). An automated method for finding molecular complexes in large protein interaction networks. BMC Bioinform..

[48] Tomato Genome C (2012). The tomato genome sequence provides insights into fleshy fruit evolution. Nature.

[49] Hatakeyama S, Jensen JP, Weissman AM (1997). Subcellular localization and ubiquitin-conjugating enzyme (E2) interactions of mammalian HECT family ubiquitin protein ligases. J. Biol. Chem..

[50] Flasza M (2006). Regulation of the nuclear localization of the human Nedd4-related WWP1 protein by Notch. Mol. Membr. Biol..

[51] Rastogi S, Liberles DA (2005). Subfunctionalization of duplicated genes as a transition state to neofunctionalization. BMC Evol. Biol..

[52] Scheffner M, Kumar S (1843). Mammalian HECT ubiquitin-protein ligases: Biological and pathophysiological aspects. Biochim. Biophys. Acta.

[53] Patil, A. *Protein–Protein Interaction Databases*. (2018).

[54] Melino G, Cecconi F, Pelicci PG, Mak TW, Bernassola F (2019). Emerging roles of HECT-type E3 ubiquitin ligases in autophagy regulation. Mol. Oncol..

[55] Miao Y, Zentgraf U (2010). A HECT E3 ubiquitin ligase negatively regulates Arabidopsis leaf senescence through degradation of the transcription factor WRKY53. Plant J..

[56] Diaz-Granados A (2020). The effector GpRbp-1 of Globodera pallida targets a nuclear HECT E3 ubiquitin ligase to modulate gene expression in the host. Mol. Plant Pathol..

[57] Bulger M, Groudine M (2011). Functional and mechanistic diversity of distal transcription enhancers. Cell.

[58] Bailey TL (2009). MEME SUITE: Tools for motif discovery and searching. Nucleic Acids Res..

[59] Xu G, Guo C, Shan H, Kong H (2012). Divergence of duplicate genes in exon-intron structure. Proc. Natl. Acad. Sci. U S A.

[60] Wright PE, Dyson HJ (1999). Intrinsically unstructured proteins: Re-assessing the protein structure-function paradigm. J. Mol. Biol..

[61] Gangavarapu V (2006). Mms2-Ubc13-dependent and -independent roles of Rad5 ubiquitin ligase in postreplication repair and translesion DNA synthesis in Saccharomyces cerevisiae. Mol. Cell Biol..

[62] Citterio E (2004). Np95 is a histone-binding protein endowed with ubiquitin ligase activity. Mol. Cell Biol..

[63] Wilkinson KD (1997). Regulation of ubiquitin-dependent processes by deubiquitinating enzymes. FASEB J..

[64] Zouine M (2017). TomExpress, a unified tomato RNA-Seq platform for visualization of expression data, clustering and correlation networks. Plant J..

[65] Miller, C. *et al.* Natural variation in expression of the HECT E3 ligase UPL3 influences seed size and crop yields in <em>Brassica napus</em> by altering regulatory gene expression. *bioRxiv*, 334581, 10.1101/334581 (2018).

[66] Finn, R. D., Clements, J. & Eddy, S. R. *HMMER Web Server: Interactive Sequence Similarity Searching*, 2011). 10.1093/nar/gkr367 PMC3125773 21593126

[67] El-Gebali S (2019). The Pfam protein families database in 2019. Nucleic Acids Res..

[68] Letunic I, Bork P (2018). 20 years of the SMART protein domain annotation resource. Nucleic Acids Res..

[69] Gasteiger, E. *et al.* in *The Proteomics Protocols Handbook* (ed John M. Walker) pp. 571–607 (Humana Press, 2005).

[70] Goodstein DM (2012). Phytozome: A comparative platform for green plant genomics. Nucleic Acids Res..

[71] Pierleoni A, Martelli PL, Fariselli P, Casadio R (2006). BaCelLo: A balanced subcellular localization predictor. Bioinformatics.

[72] Goldberg T (2014). LocTree3 prediction of localization. Nucleic Acids Res..

[73] Mi H, Muruganujan A, Ebert D, Huang X, Thomas PD (2019). PANTHER version 14: More genomes, a new PANTHER GO-slim and improvements in enrichment analysis tools. Nucleic Acids Res..

[74] Howe KL (2020). Ensembl Genomes 2020-enabling non-vertebrate genomic research. Nucleic Acids Res..

[75] Voorrips RE (2002). MapChart: Software for the graphical presentation of linkage maps and QTLs. J. Hered..

[76] WordArt.com. *Word Cloud Art Creator*, https://wordart.com (2009).

[77] Bailey TL, Gribskov M (1998). Combining evidence using p-values: Application to sequence homology searches. Bioinformatics.

[78] Lu S (2020). CDD/SPARCLE: The conserved domain database in 2020. Nucleic Acids Res..

[79] Huerta-Cepas J (2017). Fast genome-wide functional annotation through orthology assignment by eggNOG-mapper. Mol. Biol. Evol..

[80] Huerta-Cepas J (2019). eggNOG 5.0: A hierarchical, functionally and phylogenetically annotated orthology resource based on 5090 organisms and 2502 viruses. Nucleic Acids Res..

[81] Gao T (2013). UUCD: A family-based database of ubiquitin and ubiquitin-like conjugation. Nucleic Acids Res..

[82] McLeay RC, Bailey TL (2010). Motif Enrichment Analysis: A unified framework and an evaluation on ChIP data. BMC Bioinform..

[83] Hu B (2015). GSDS 2.0: An upgraded gene feature visualization server. Bioinformatics.

[84] Berman HM (2000). The protein data bank. Nucleic Acids Res..

[85] Waterhouse AM, Procter JB, Martin DM, Clamp M, Barton GJ (2009). Jalview Version 2–a multiple sequence alignment editor and analysis workbench. Bioinformatics.

[86] Crooks GE, Hon G, Chandonia JM, Brenner SE (2004). WebLogo: A sequence logo generator. Genome Res..

[87] Schneider TD, Stephens RM (1990). Sequence logos: A new way to display consensus sequences. Nucleic Acids Res..

[88] Pettersen EF (2004). UCSF Chimera–a visualization system for exploratory research and analysis. J. Comput. Chem..

[89] Laskowski RA, MacArthur MW, Moss DS, Thornton JM (1993). PROCHECK: A program to check the stereochemical quality of protein structures. J. Appl. Crystallogr..

[90] Bowie JU, Luthy R, Eisenberg D (1991). A method to identify protein sequences that fold into a known three-dimensional structure. Science.

[91] Colovos C, Yeates TO (1993). Verification of protein structures: Patterns of nonbonded atomic interactions. Protein Sci..

[92] Benkert P, Biasini M, Schwede T (2011). Toward the estimation of the absolute quality of individual protein structure models. Bioinformatics.

[93] Kumar S, Stecher G, Li M, Knyaz C, Tamura K (2018). MEGA X: Molecular evolutionary genetics analysis across computing platforms. Mol. Biol. Evol..

[94] Letunic I, Bork P (2019). Interactive Tree Of Life (iTOL) v4: Recent updates and new developments. Nucleic Acids Res..

[95] Szklarczyk D (2019). STRING v11: Protein-protein association networks with increased coverage, supporting functional discovery in genome-wide experimental datasets. Nucleic Acids Res..

[96] Shannon P (2003). Cytoscape: A software environment for integrated models of biomolecular interaction networks. Genome Res..

[97] Gotz S (2008). High-throughput functional annotation and data mining with the Blast2GO suite. Nucleic Acids Res..

[98] UniProt C (2019). UniProt: A worldwide hub of protein knowledge. Nucleic Acids Res..

[99] Moriya Y, Itoh M, Okuda S, Yoshizawa AC, Kanehisa M (2007). KAAS: An automatic genome annotation and pathway reconstruction server. Nucleic Acids Res..

