# Supplementary material for: Genome-wide analysis of HECT E3 ubiquitin ligase gene family in Solanum lycopersicum
Source: Sci Rep. 2021 Aug 5;11:15891. doi: 10.1038/s41598-021-95436-2 (PMC8342558; doi:10.1038/s41598-021-95436-2)
Supplement: Supplementary file 1 — Supplementary Information. [file 41598_2021_95436_MOESM1_ESM.docx]

**Genome-wide analysis of HECT E3 ubiquitin ligase gene family in *Solanum lycopersicum***

Bhaskar Sharma^1,2*^, Harshita Saxena^2^, and Harshita Negi^2^

^1^School of Life and Environmental Sciences, Faculty of Science, Engineering, and Built Environment, Deakin University, Geelong, VIC-3220, Australia

^2^Department of Biotechnology, TERI School of Advanced Studies, New Delhi, India 110070

^*^Correspondence:

Bhaskar Sharma

bs211190@yahoo.com

**SUPPLEMENTARY INFORMATION**

| **E3 Ubiquitin Ligase** | **Gene Name** | **HECT Domain** | **Mw** | **pI** | **Amino Acids** | **Introns** | **Exons** | **Instability Index** | **Aliphatic Index** | **GRAVY** | **Gene size** | **Chromosomal Position** | **Sub-cellular localization**  **(BaCelLo/ LocTree3)** |
| --- | --- | --- | --- | --- | --- | --- | --- | --- | --- | --- | --- | --- | --- |
| **Solyc01g057900.2.1** | *SlHECT1* | 723 - 1030 | 117955.47 | 6.3 | 1030 | 25 | 26 | 47.19 | 94.02 | -0.144 | 37148 | SL2.50ch01:64141356-64178503 | Nucleus/ Nucleus |
| **Solyc01g111530.2.1** | *SlHECT2* | 1492 - 1860 | 199910.08 | 5.74 | 1860 | 18 | 19 | 48.1 | 86.12 | -0.291 | 11273 | SL2.50ch01:97755719-97766991 | Chloroplast/ Nucleus |
| **Solyc04g076620.2.1** | *SlHECT3* | 3288 - 3600 | 397251.33 | 5 | 3600 | 17 | 18 | 50.28 | 89.34 | -0.303 | 17802 | SL2.50ch04:61503203-61521004 | Nucleus/Cytoplasm |
| **Solyc05g054080.2.1** | *SlHECT4* | 579- 890 | 101652.29 | 5.89 | 891 | 2 | 3 | 45.01 | 85.02 | -0.233 | 7835 | SL2.50ch05:64056582-64064416 | Nucleus/Cytoplasm |
| **Solyc07g065630.2.1** | *SlHECT5* | 3455- 3757 | 412777.85 | 4.99 | 3757 | 16 | 17 | 47.26 | 91.91 | -0.222 | 16245 | SL2.50ch07:67317838-67334082 | Nucleus/Cytoplasm |
| **Solyc09g005150.1.1** | *SlHECT6* | 527- 839 | 96532.75 | 6.31 | 839 | 2 | 3 | 40.64 | 92.57 | -0.158 | 3981 | SL2.50ch09:145682-149662 | Chloroplast/Cytoplasm |
| **Solyc09g005160.1.1** | *SlHECT7* | 517 - 829 | 95739.23 | 6.1 | 829 | 2 | 3 | 42.47 | 87.58 | -0.172 | 4278 | SL2.50ch09:151592-155869 | Nucleus/Cytoplasm |
| **Solyc09g007310.2.1** | *SlHECT8* | 1204 - 1553 | 173042.14 | 5.67 | 1553 | 16 | 17 | 47.77 | 92.55 | -0.178 | 9286 | SL2.50ch09:907686-916971 | Nucleus/ Nucleus |
| **Solyc09g008700.1.1** | *SlHECT9* | 846 - 1141 | 129255.02 | 8.3 | 1144 | 13 | 14 | 42.37 | 96.28 | -0.128 | 11770 | SL2.50ch09:2135770-2147539 | Chloroplast/Cytoplasm |
| **Solyc09g056040.2.1** | *SlHECT10* | 127 - 355 | 42819.06 | 5.85 | 363 | 5 | 6 | 44.98 | 81.57 | -0.466 | 3841 | SL2.50ch09:46730636-46734476 | Nucleus/Cytoplasm |
| **Solyc10g055450.1.1** | *SlHECT11* | 1476 - 1846 | 199865.85 | 5.73 | 1846 | 16 | 17 | 49.34 | 85.58 | -0.264 | 12062 | SL2.50ch10:56774992-56787053 | Chloroplast/Nucleus |
| **Solyc10g083470.1.1** | *SlHECT12* | 112 - 423 | 48770.98 | 5.48 | 423 | 2 | 3 | 43.83 | 84.11 | -0.145 | 1810 | SL2.50ch10:63257603-63259412 | Cytoplasm/ Cytoplasm |
| **Solyc11g012330.1.1** | *SlHECT13* | 1 - 285 | 33008.62 | 4.65 | 286 | 2 | 3 | 40.04 | 89.27 | -0.066 | 1383 | SL2.50ch11:5182505-5183887 | Nucleus/Cytoplasm |
| **Solyc12g094560.1.1** | *SlHECT14* | 490 - 800 | 93048.77 | 5.51 | 801 | 2 | 3 | 48.72 | 90.5 | -0.2 | 2576 | SL2.50ch12:64756119-64758694 | Chloroplast/Cytoplasm |

**Supplementary Table S1:** The physicochemical parameters (molecular weight (Mw), iso-electric point (pI), number of amino acids, instability index, aliphatic index, and grand average of hydropathicity (GRAVY), gene size, introns, exons, chromosomal position, HECT domain, and sub-cellular localization) details of 14 identified HECT E3 ubiquitin ligases in tomato.

| **S.NO.** | **GENE**  **NAME** | **HORMONES** | | | | | **DEFENSE AND**  **STRESS RESPONSIVENESS** | **ABIOTIC STRESS** | | | | | **PLANT DEVELOPMENT** | | | | | |
| --- | --- | --- | --- | --- | --- | --- | --- | --- | --- | --- | --- | --- | --- | --- | --- | --- | --- | --- |
|  |  | **Auxin** | **Gibberellin** | **Abscisic Acid** | **Salicyclic Acid** | **Methyl Jasmonate** |  | **Light responsiveness** | **Drought Inducibility** | **Low temperature** | **Low- oxygen response**  **(Anaerobic induction)** | **No oxygen response (Anoxic-specific induction)** | **Meristem expression** | **Endosperm expression** | **Differentiation of Palisade mesophyll cells** | **Zein metabolism expression** | **Seed-specific regulation** | **Circadian control** |
|  | *SlHECT1* | ✔ | ✔ | ✔ |  |  | ✔ | ✔ |  | ✔ |  |  |  |  |  |  |  |  |
|  | *SlHECT2* |  | ✔ | ✔ |  | ✔ | ✔ | ✔ | ✔ | ✔ | ✔ |  |  |  |  |  |  |  |
|  | *SlHECT3* |  | ✔ | ✔ |  |  | ✔ | ✔ | ✔ | ✔ |  | ✔ |  |  |  | ✔ |  |  |
|  | *SlHECT4* | ✔ | ✔ | ✔ |  |  |  | ✔ |  | ✔ |  | ✔ | ✔ |  |  |  |  |  |
|  | *SlHECT5* | ✔ | ✔ | ✔ |  |  | ✔ | ✔ |  | ✔ | ✔ |  |  |  | ✔ |  |  |  |
|  | *SlHECT6* | ✔ |  | ✔ |  | ✔ |  | ✔ |  |  | ✔ |  |  | ✔ |  |  |  |  |
|  | *SlHECT7* | ✔ | ✔ | ✔ |  | ✔ | ✔ | ✔ |  | ✔ | ✔ |  |  | ✔ | ✔ | ✔ | ✔ |  |
|  | *SlHECT8* |  |  |  |  |  | ✔ | ✔ | ✔ | ✔ |  | ✔ | ✔ |  |  |  |  | ✔ |
|  | *SlHECT9* |  |  | ✔ | ✔ |  | ✔ | ✔ |  | ✔ |  |  | ✔ | ✔ | ✔ |  |  |  |
|  | *SlHECT10* |  |  |  |  | ✔ |  | ✔ | ✔ |  | ✔ |  | ✔ |  |  | ✔ |  |  |
|  | *SlHECT11* |  | ✔ | ✔ |  |  | ✔ | ✔ |  |  | ✔ |  |  |  |  |  |  |  |
|  | *SlHECT12* |  |  | ✔ | ✔ | ✔ |  | ✔ |  |  | ✔ |  |  |  |  |  |  |  |
|  | *SlHECT13* |  | ✔ | ✔ |  | ✔ |  | ✔ | ✔ | ✔ | ✔ |  | ✔ |  |  | ✔ |  | ✔ |
|  | *SlHECT14* |  | ✔ |  | ✔ | ✔ | ✔ | ✔ | ✔ |  | ✔ |  |  |  |  |  |  | ✔ |

**Supplementary Table S2:** The role of different promoter elements of HECT gene family members in tomato plant development and external responses.

| **S.No.** | **UUCD ID** | **Chromosomal Location** | **Nomenclature** |
| --- | --- | --- | --- |
| ***Arabidopsis thaliana*** | | | |
|  | UUC-ArT-01454 | Chr1:20879466-20895393 | *AtHECT1* |
|  | UUC-ArT-01458 | Chr1:26488522-26501281 | *AtHECT2* |
|  | UUC-ArT-01468 | Chr3:5873253-5881655 | *AtHECT3* |
|  | UUC-ArT-01543 | Chr3:19679208-19685360 | *AtHECT4* |
|  | UUC-ArT-01564 | Chr4:7445362-7449086 | *AtHECT5* |
|  | UUC-ArT-01443 | Chr4:18041031-18049292 | *AtHECT6* |
|  | UUC-ArT-01534 | Chr5:662064-669107 | *AtHECT7* |
| ***Oryza sativa*** | | | |
| 1. | UUC-OrS-00166 | Chr2:86464-91606 | *OsHECT1* |
| 2. | UUC-OrS-00168 | Chr3:27257668-27263489 | *OsHECT2* |
| 3. | UUC-OrS-00167 | Chr5:1223963-1230057 | *OsHECT3* |
| 4. | UUC-OrS-00814 | Chr5:3472305-3485106 | *OsHECT4* |
| 5. | UUC-OrS-00849 | Chr5:22776036-22779105 | *OsHECT5* |
| 6. | UUC-OrS-00165 | Chr9:4016486-4036019 | *OsHECT6* |
| 7. | UUC-OrS-00169 | Chr9:18687740-18694228 | *OsHECT7* |
| 8. | UUC-OrS-00595 | Chr12:13697068-13702579 | *OsHECT8* |
| ***Populus trichocarpa*** | | | |
|  | UUC-PoT-00246 | Chr1:36787976-36798515 | *PtHECT1* |
|  | UUC-PoT-00247 | Chr2:8127368-8140539 | *PtHECT2* |
|  | UUC-PoT-00250 | Chr4:18064665-18074092 | *PtHECT3* |
|  | UUC-PoT-01167 | Chr6:771374-778010 | *PtHECT4* |
|  | UUC-PoT-00249 | Chr6:10615219-10622852 | *PtHECT5* |
|  | UUC-PoT-00252 | Chr8:6276704-6290951 | *PtHECT6* |
|  | UUC-PoT-00251 | Chr9:10847780-10857668 | *PtHECT7* |
|  | UUC-PoT-01283 | Chr10:15060830-15075832 | *PtHECT8* |
|  | UUC-PoT-00248 | Chr11:12018356-12033619 | *PtHECT9* |
|  | UUC-PoT-00254 | Chr16:646953-650723 | *PtHECT10* |
|  | UUC-PoT-00256 | Chr16:653317-653955 | *PtHECT11* |
|  | UUC-PoT-00253 | Chr16:6448469-6457455 | *PtHECT12* |
|  | UUC-PoT-00255 | Chr16:9400821-9406684 | *PtHECT13* |
|  | UUC-PoT-00257 | Chr16:9408711-9414353 | *PtHECT14* |
| ***Vitis vinifera*** | | | |
|  | UUC-ViV-00922 | Chr3:1612568-1639214 | *VvHECT1* |
|  | UUC-ViV-00452 | Chr4:20328806-20339108 | *VvHECT2* |
|  | UUC-ViV-00143 | Chr5:271696-308068 | *VvHECT3* |
|  | UUC-ViV-00654 | Chr5:2164802-2167281 | *VvHECT4* |
|  | UUC-ViV-00798 | Chr8:14214405-14226967 | *VvHECT5* |
|  | UUC-ViV-00238 | Chr8:18237734-18264946 | *VvHECT6* |
|  | UUC-ViV-00814 | Chr9:12740901-12741553 | *VvHECT7* |
|  | UUC-ViV-00508 | Chr18:6235224-6255464 | *VvHECT8* |
|  | UUC-ViV-00960 | Chr19:8744183-8753166 | *VvHECT9* |
|  | UUC-ViV-00807 | Un:9089113-9109930 | *VvHECT10* |
| ***Sorghum bicolor*** | | | |
|  | UUC-SoB-00121 | Chr1:10695999-10704819 | *SbHECT1* |
|  | UUC-SoB-00025 | Chr2:37105943-37120815 | *SbHECT2* |
|  | UUC-SoB-00026 | Chr2:63377624-63378097 | *SbHECT3* |
|  | UUC-SoB-00357 | Chr2:63379346-63382938 | *SbHECT4* |
|  | UUC-SoB-00670 | Chr4:137111-142639 | *SbHECT5* |
|  | UUC-SoB-01040 | Chr6:6984829-6995313 | *SbHECT6* |
|  | UUC-SoB-01222 | Chr8:33992247-34008557 | *SbHECT7* |
|  | UUC-SoB-01358 | Chr9:2255916-2262742 | *SbHECT8* |
|  | UUC-SoB-01385 | Chr9:5504353-5518561 | *SbHECT9* |
|  | UUC-SoB-01362 | Chr9:52463776-52472136 | *SbHECT10* |
| ***Zea mays*** | | | |
|  | UUC-ZeM-00259 | Chr2:83539268-83558706 | *ZmHECT1* |
|  | UUC-ZeM-00258 | Chr2:155072701-155102547 | *ZmHECT2* |
|  | UUC-ZeM-01481 | Chr2:189501893-189517266 | *ZmHECT3* |
|  | UUC-ZeM-00256 | Chr3:111553578-111570930 | *ZmHECT4* |
|  | UUC-ZeM-00263 | Chr5:13576263-13591781 | *ZmHECT5* |
|  | UUC-ZeM-00260 | Chr5:66431525-66441796 | *ZmHECT6* |
|  | UUC-ZeM-01517 | Chr5:7141233-7153467 | *ZmHECT7* |
|  | UUC-ZeM-00262 | Chr6:125545108-125554361 | *ZmHECT8* |
|  | UUC-ZeM-00266 | Chr6:125824374-125827493 | *ZmHECT9* |
|  | UUC-ZeM-00265 | Chr6:28466440-28477852 | *ZmHECT10* |
|  | UUC-ZeM-01319 | Chr7:132186216-132203350 | *ZmHECT11* |
|  | UUC-ZeM-00261 | Chr8:117746358-117761424 | *ZmHECT12* |
|  | UUC-ZeM-00257 | Chr10:20699314-20716048 | *ZmHECT13* |
|  | UUC-ZeM-00264 | Chr10:51680731-51685636 | *ZmHECT14* |
| ***Mus musculus*** | | | |
|  | UUC-MuM-00498 | Chr1:53863726-54251878 | *MmHECT1* |
|  | UUC-MuM-00248 | Chr1:84717764-84835879 | *MmHECT2* |
|  | UUC-MuM-00042 | Chr2:154959245-155052591 | *MmHECT3* |
|  | UUC-MuM-00039 | Chr4:19535450-19636140 | *MmHECT4* |
|  | UUC-MuM-00388 | Chr4:116667922-116677882 | *MmHECT5* |
|  | UUC-MuM-00578 | Chr5:29895782-30002617 | *MmHECT6* |
|  | UUC-MuM-00976 | Chr5:114830637-114871177 | *MmHECT7* |
|  | UUC-MuM-00167 | Chr5:121670228-121818586 | *MmHECT8* |
|  | UUC-MuM-00060 | Chr5:145637371-145726716 | *MmHECT9* |
|  | UUC-MuM-00215 | Chr6:57530986-57615130 | *MmHECT10* |
|  | UUC-MuM-00116 | Chr6:58783694-58870392 | *MmHECT11* |
|  | UUC-MuM-00423 | Chr7:63305525-63487164 | *MmHECT12* |
|  | UUC-MuM-00433 | Chr7:66484122-66562097 | *MmHECT13* |
|  | UUC-MuM-00064 | Chr8:109960298-110082494 | *MmHECT14* |
|  | UUC-MuM-00179 | Chr9:66198333-66356582 | *MmHECT15* |
|  | UUC-MuM-00012 | Chr9:72510364-72597649 | *MmHECT16* |
|  | UUC-MuM-00384 | Chr10:45297635-45432151 | *MmHECT17* |
|  | UUC-MuM-00518 | Chr10:62706659-62780622 | *MmHECT18* |
|  | UUC-MuM-00079 | Chr11:106681380-106782029 | *MmHECT19* |
|  | UUC-MuM-00448 | Chr12:52449048-52477973 | *MmHECT20* |
|  | UUC-MuM-00230 | Chr12:52844709-52930523 | *MmHECT21* |
|  | UUC-MuM-00742 | Chr12:86259100-86311850 | *MmHECT22* |
|  | UUC-MuM-00773 | Chr13:14318705-14615493 | *MmHECT23* |
|  | UUC-MuM-00183 | Chr15:37897083-38008608 | *MmHECT24* |
|  | UUC-MuM-00738 | Chr18:65047410-65377480 | *MmHECT25* |
|  | UUC-MuM-00730 | Chr19:36629129-36695625 | *MmHECT26* |
|  | UUC-MuM-00561 | ChrX:148237870-148369960 | *MmHECT27* |
| ***Homo sapiens*** | | | |
|  | UUC-HoS-00146 | Chr1:45468212-45477001 | *HsHECT1* |
|  | UUC-HoS-01266 | Chr2:197063977-197457335 | *HsHECT2* |
|  | UUC-HoS-01060 | Chr2: 229763838-229923239 | *HsHECT3* |
|  | UUC-HoS-00123 | Chr2:230631930-230786725 | *HsHECT4* |
|  | UUC-HoS-00211 | Chr4:89299891-89364249 | *HsHECT5* |
|  | UUC-HoS-00422 | Chr4:89378268-89427314 | *HsHECT6* |
|  | UUC-HoS-00126 | Chr4:89444961-89570998 | *HsHECT7* |
|  | UUC-HoS-00189 | Chr6:83602117-83775560 | *HsHECT8* |
|  | UUC-HoS-00219 | Chr6:105175968-105307794 | *HsHECT9* |
|  | UUC-HoS-00180 | Chr7:43152198-43602938 | *HsHECT10* |
|  | UUC-HoS-00380 | Chr7:98625064-98741723 | *HsHECT11* |
|  | UUC-HoS-00131 | Chr7:156931607-157062066 | *HsHECT12* |
|  | UUC-HoS-00358 | Chr8:87354967-87480732 | *HsHECT13* |
|  | UUC-HoS-00141 | Chr10:69681665-69835040 | *HsHECT14* |
|  | UUC-HoS-00152 | Chr10:93170096-93274518 | *HsHECT15* |
|  | UUC-HoS-00182 | Chr12:109915207-109974507 | *HsHECT16* |
|  | UUC-HoS-00465 | Chr12:112597992-112819896 | *HsHECT17* |
|  | UUC-HoS-00181 | Chr14:31028329-31089269 | *HsHECT18* |
|  | UUC-HoS-00446 | Chr14:31569324-31677010 | *HsHECT19* |
|  | UUC-HoS-00534 | Chr14:75127955-75179818 | *HsHECT20* |
|  | UUC-HoS-00094 | Chr15:25582381-25684128 | *HsHECT21* |
|  | UUC-HoS-00052 | Chr15:28356186-28567298 | *HsHECT22* |
|  | UUC-HoS-00073 | Chr15:56119120-56285835 | *HsHECT23* |
|  | UUC-HoS-00132 | Chr15:63900818-64126147 | *HsHECT24* |
|  | UUC-HoS-00006 | Chr16:69796209-69975644 | *HsHECT25* |
|  | UUC-HoS-00379 | Chr17:62540735-62658386 | *HsHECT26* |
|  | UUC-HoS-00316 | Chr18:55711619-56068772 | *HsHECT27* |
|  | UUC-HoS-00306 | Chr20:32951041-33099198 | *HsHECT28* |
|  | UUC-HoS-00191 | ChrX:53559105-53713673 | *HsHECT29* |

**Supplementary Table S3:** Nomenclature of the HECT protein sequences of *Arabidopsis thaliana*, *Oryza sativa*, *Populus trichocarpa, Vitis vinifera, Sorghum bicolor, Zea mays, Mus musculus*, and *Homo sapiens* on the basis of their chromosomal location.

| **Scanned Motifs** | | | |
| --- | --- | --- | --- |
| **Motif Number** | **Width** | **Tomato Motif Sequence** | **Best Possible Match** |
| 1 | 21 | FWEIVGSFSAEQRNALLFFVT | FWEIVGSFSAEQRNALLFFVT |
| 2 | 75 | FFPNPASKVDPLHLEYFTFSGRVIALALLHKIQIGIVFDRVFFLQLAGEDISLEDIRDADPYLYKSCKEILEMDP | FFPNPASKVDPLHLEYFTFSGRVIALALLHKIQIGIVFDRVFFLQLAGEDISLEDIRDADPDLYSSCKEILEMDP |
| 3 | 76 | EELYEMLIDRSRLLEESFEYIGHASPKSLRGQLFIQFENEEATGPGVLREWFSLVCEAIFNPQNALFVACPNDGRR | EELYEMLIDRSRLLEESFEYIGHASPRSLRGQLFIQFENEEATGPGVLREWFSLVCEAIFNPQNALFVSCPNDGRR |
| 4 | 41 | CPNGKNTRVNSENREEYVBLVVDHRFVTSIAQQVAAFASGF | CPNGKDTRVDSENREEYVBLVVDHRFVTSIAPQVAAFASGF |
| 5 | 39 | DDHLPSSHTCFNYLKLPPYPSKEIMQERLLIIIQEGVGC | DDHLPSSHTCFNYLKLPPYPSKEIMQERLLIAIQEGVGC |
| 6 | 193 | SIGTEDSLSTFSVDSFVPVLVGLLNHESNPDIMLLAARALTHLVDVLPSSCAAVVHYGAVSCFVARLLTIEYMDLAEQSLQALKKISQEHPTACLQAGALMAVLSYLDFFSTGVQRVALATAANMCKKLPSDAPDFVMEAVPLLTNLLQYHDAKVLEHASICLTRIAEAFASSPEKLDELCNHGLVTQAASLI | SIGTEDSLSTFSVDSFVPVLVGLLNHESNPDIMLLAARALTHLVDVLPSSCAAVVHYGAVSCFVARLLTIEYMDLAEQSLQALKKISQEDPTACLQAGALMAVLSYLDFFSTGVQRVALATAANMCKKLPSDASDFVMEAVPLLTBLLQYHDAKVLEHASICLTRIAEAFASSPEKLDZLCNHGLVTQAASLI |
| 7 | 22 | GSKTAISVEDLKAHTDYNGYYT | GSLTAISVEDLKAHTDYNGYYT |
| 8 | 57 | MEEMFWZKMRQRKVSLCFLIVRFAKKSEDYRWILEHKEVTNFKVRRHFALMMLPEGR | SEEMFWEKMRQRKVSLCFLIVRFAKKSEDYRWILEHKEVTNFKVRRRFALMMLPEVR |
| 9 | 120 | QGELPCDLEKSNPTYBILYLLRVLEGLNQLAPRLRVQSVIDDFSEGKISSLDELGTTGIKIPSEEFVNSKLTPKLARQIQDALALCSGSLPSWCYQLTKSCPFLFPFETRRQYFYSTAFG | QGELPCDLEKSNPTYSILALLRVLEGLNQLAPRLGVLSVJDDFSEGKILSLDELGTTGSKJPSEEFVNSKLTPKLARQIQDALALCSGSLPSWCSQLTRSCPFLFPFETRRQYFYSTAFG |
| 10 | 21 | SIKRLPLEGFAGLDPKLTIHR | SIKRLPLEGFAGLDSKLTIVR |
| **Motif Enrichment Analysis** | | | |
| **Enriched tomato motif in *Arabidopsis thaliana*** | | | **Enriched tomato motif in *Populus trichocarpa*** |
| SIKRLPLEGFAGLDSKLTIVR | | | SIKRLPLEGFAGLDSKLTIVR |
| GSLTAISVEDLKAHTDYNGYYT | | | GSLTAISVEDLKAHTDYNGYYT |
| CPNGKDTRVDSENREEYVBLVVDHRFVTSIAPQVAAFASGF | | | DDHLPSSHTCFNYLKLPPYPSKEIMQERLLIATQEGVGC |
| FWEIVGSFSAEQRNALLFFVT | | | FWEIVGSFSAEQRNALLFFVT |
| DDHLPSSHTCFNYLKLPPYPSKEIMQERLLIATQEGVGC | | | CPNGKDTRVDSENREEYVBLVVDHRFVTSIAPQVAAFASGF |
| **Enriched tomato motif in *Oryza sativa*** | | | **Enriched tomato motif in *Sorghum bicolor*** |
| SIKRLPLEGFAGLDSKLTIVR | | | SIKRLPLEGFAGLDSKLTIVR |
| DDHLPSSHTCFNYLKLPPYPSKEIMQERLLIATQEGVGC | | | GSLTAISVEDLKAHTDYNGYYT |
| CPNGKDTRVDSENREEYVBLVVDHRFVTSIAPQVAAFASGF | | | DDHLPSSHTCFNYLKLPPYPSKEIMQERLLIATQEGVGC |
| FWEIVGSFSAEQRNALLFFVT | | | FWEIVGSFSAEQRNALLFFVT |
| GSLTAISVEDLKAHTDYNGYYT | | | CPNGKDTRVDSENREEYVBLVVDHRFVTSIAPQVAAFASGF |
| **Enriched tomato motif in *Vitis vinifera*** | | | **Enriched tomato motif in *Zea Mays*** |
| SIKRLPLEGFAGLDSKLTIVR | | | SIKRLPLEGFAGLDSKLTIVR |
| CPNGKDTRVDSENREEYVBLVVDHRFVTSIAPQVAAFASGF | | | DDHLPSSHTCFNYLKLPPYPSKEIMQERLLIAIQEGVGC |
| FWEIVGSFSAEQRNALLFFVT | | | FWEIVGSFSAEQRNALLFFVT |
| GSLTAISVEDLKAHTDYNGYYT | | | CPNGKDTRVDSENREEYVBLVVDHRFVTSIAPQVAAFASGF |
| DDHLPSSHTCFNYLKLPPYPSKEIMQERLLIAIQEGVGC | | | GSLTAISVEDLKAHTDYNGYYT |
| **Enriched tomato motif in *Homo sapiens*** | | | **Enriched tomato motif in *Mus musculus*** |
| FWEIVGSFSAEQRNALLFFVT | | | FWEIVGSFSAEQRNALLFFVT |
| DDHLPSSHTCFNYLKLPPYPSKEIMQERLLIATQEGVGC | | | DDHLPSSHTCFNYLKLPPYPSKEIMQERLLIATQEGVGC |
| SIKRLPLEGFGGLDSKLTIVR | | | SIKRLPLEGFGGLDSKLTIVR |
| CPNGKDTRVDSENREEYVBLVVDHRFVTSIAPQVAAFASGF | | | GSLTAISVEDLKAHTDYNGYYT |
| GSLTAISVEDLKAHTDYNGYYT | | | CPNGKDTRVDSENREEYVBLVVDHRFVTSIAPQVAAFASGF |

**Supplementary Table S4:** The ten identified conserved motifs for 14 HECT E3 ligases of tomato and enriched motifs in *Arabidopsis thaliana*, *Oryza sativa*, *Populus trichocarpa, Vitis vinifera, Sorghum bicolor, Zea mays, Homo sapiens*, and *Mus musculus*.


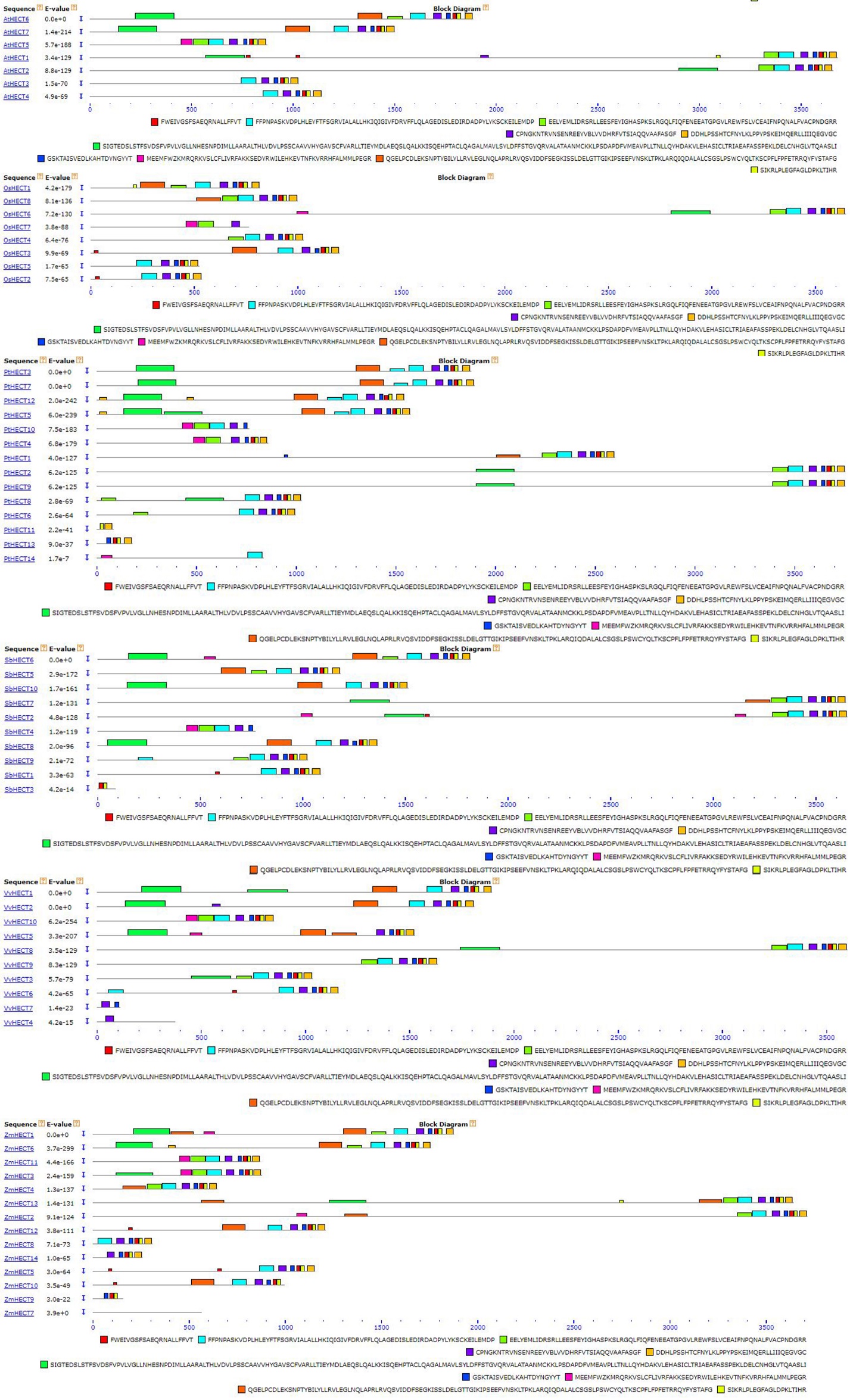


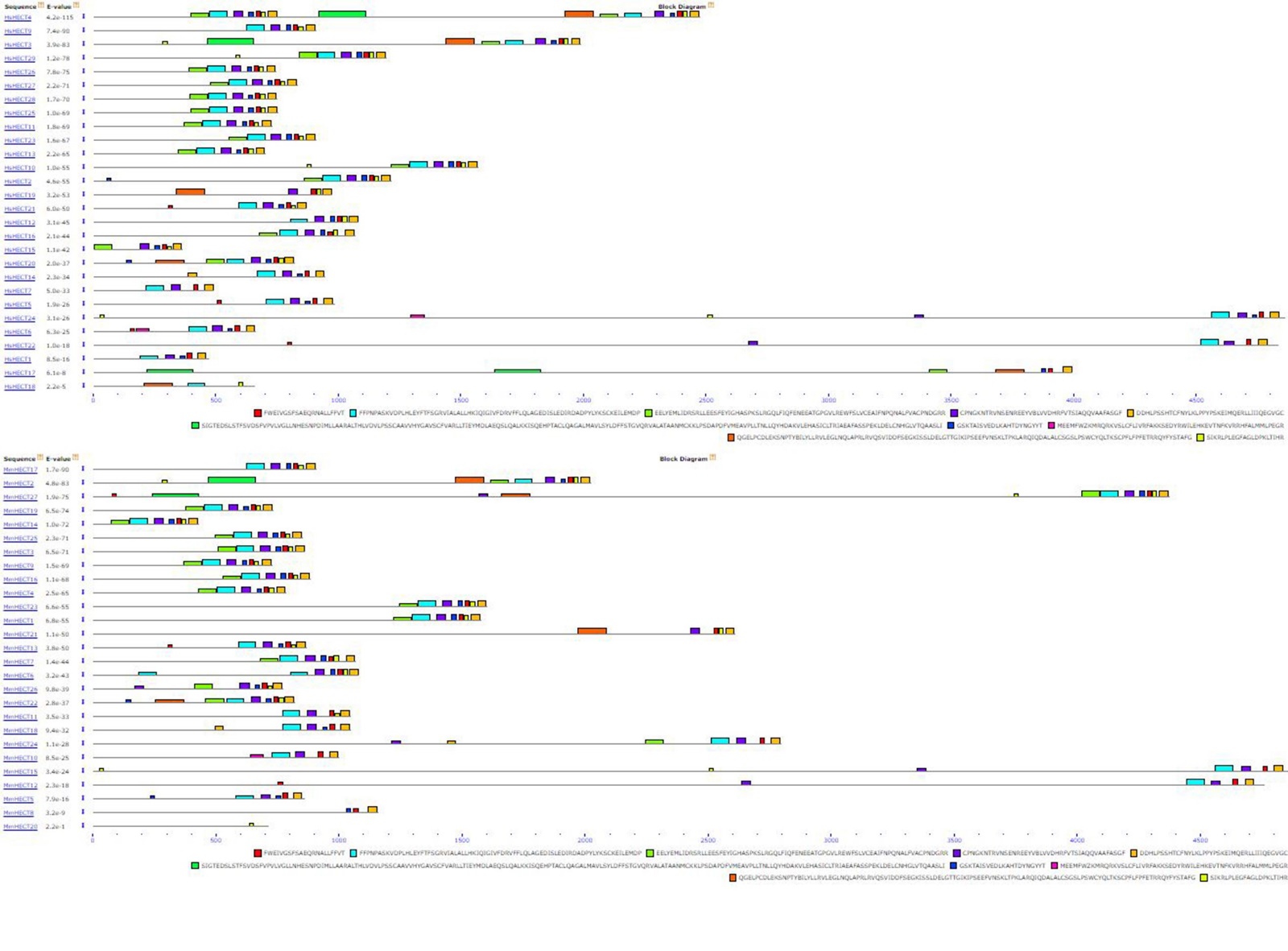


**Supplementary Figure S5**: The organisation of discovered tomato motifs in reference organisms, Arabidopsis thaliana, Oryza sativa, Populus trichocarpa, Vitis vinifera, Sorghum bicolor, Zea mays, Homo sapiens, and Mus musculus.


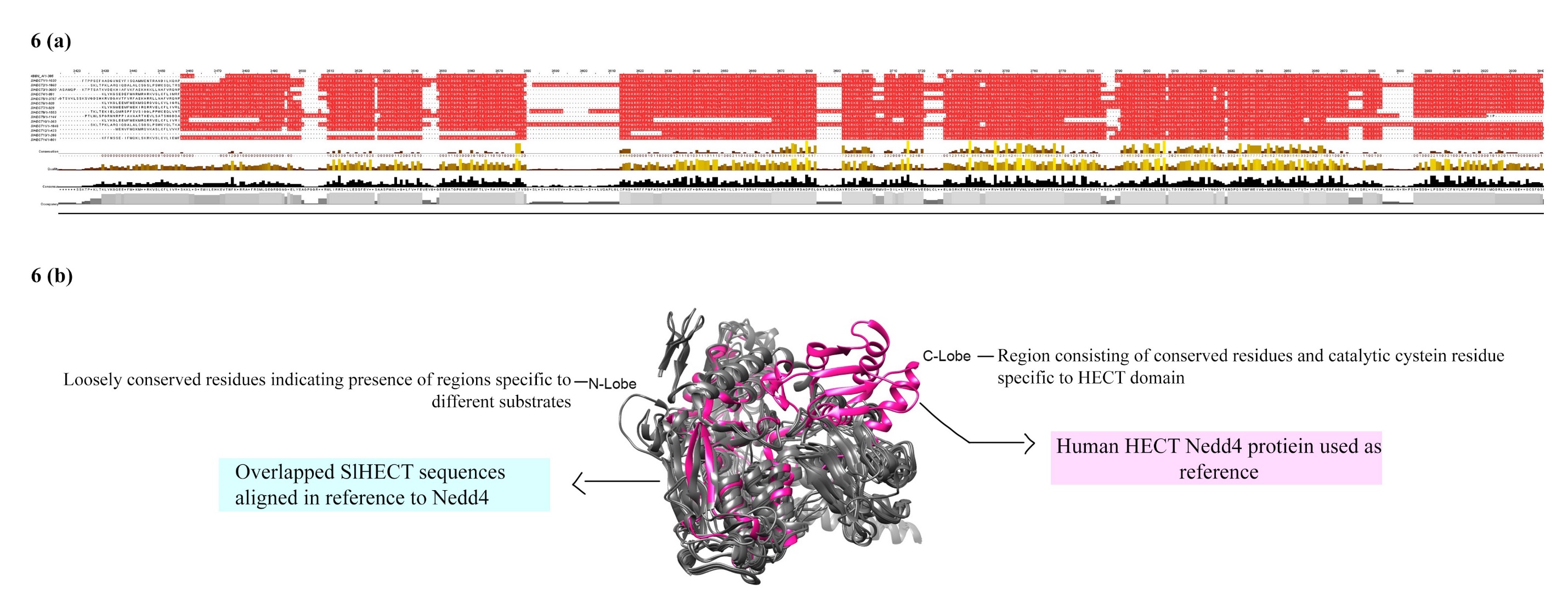


**Supplementary Figure S6:** **(a)** Multiple sequence alignment (MSA) of 14 HECT gene sequences of tomato with reference sequence of human HECT Nedd4, performed using Jalview; **(b)** 3D representation of aligned HECT domain sequences with reference sequence of human HECT Nedd4 protein. The grey color represents sequences from tomato HECT gene family and pink is representative of the Nedd4 sequence.

| **S.No.** | ***SlHECT* Member** | **Template** | **Confidence**  **(in %)** | **Sequence identity**  **(in %)** | **Percentage of α-helices**  **(in %)** | **Percentage of β-strand**  **(in %)** | **Percentage of disordered region**  **(in %)** | **Percentage of TM helix**  **(in %)** |
| --- | --- | --- | --- | --- | --- | --- | --- | --- |
|  | *SlHECT1* | c5lp8A_ | 100 | 33 | 63 | 4 | 29 | - |
|  | *SlHECT2* | c5tj7D_ | 100 | 27 | 47 | 3 | 44 | 1 |
|  | *SlHECT3* | c5lp8A_ | 100 | 53 | 53 | 1 | 45 | - |
|  | *SlHECT4* | c5lp8A_ | 100 | 31 | 61 | 4 | 31 | 2 |
|  | *SlHECT5* | c5lp8A_ | 100 | 54 | 53 | 1 | 48 | 2 |
|  | *SlHECT6* | c5lp8A_ | 100 | 31 | 60 | 6 | 21 | 4 |
|  | *SlHECT7* | c5lp8A_ | 100 | 31 | 60 | 6 | 18 | 2 |
|  | *SlHECT8* | c5tj7D_ | 100 | 28 | 51 | 3 | 39 | - |
|  | *SlHECT9* | c5lp8A_ | 100 | 29 | 66 | 4 | 25 | 9 |
|  | *SlHECT10* | c5lp8A_ | 100 | 28 | 56 | 11 | 18 | - |
|  | *SlHECT11* | c5tj7D_ | 100 | 28 | 47 | 3 | 46 | 3 |
|  | *SlHECT12* | c5lp8A_ | 100 | 29 | 51 | 11 | 11 | 4 |
|  | *SlHECT13* | c5lp8A_ | 100 | 32 | 51 | 9 | 10 | 6 |
|  | *SlHECT14* | c5lp8A_ | 100 | 30 | 59 | 8 | 21 | 2 |

**Supplementary Table S*7*:** Structural features of HECT protein models of tomato, predicted using Phyre2. Details of template used, the percentage of sequence identity and confidence of model predicted are tabulated along with the secondary structure components.

| ***SlHECT* Member** | **RAMACHANDRAN PLOT STATISTICS (%)** | | | | **VERIFY**  **3-D** | **ERRAT**  **QUALITY FACTOR** | **QMEAN**  **Z-SCORE** |
| --- | --- | --- | --- | --- | --- | --- | --- |
|  | **Most favoured regions** | **Additional allowed regions** | **Generously allowed regions** | **Disallowed regions** |  |  |  |
| *SlHECT1* | 90.71% | 6.90% | 0.80% | 1.60% | 83.18% | 68.6893 | -4.85 |
| *SlHECT2* | 82.80% | 13.70% | 1.00% | 2.50% | 36.67 | 61.3793 | -9.33 |
| *SlHECT3* | 94.30% | 5.40% | 0.00% | 0.30% | 46.30% | 94.4444 | -1.69 |
| *SlHECT4* | 90.10% | 8.60% | 0.50% | 0.80% | 73.70% | 59.3674 | -4.11 |
| *SlHECT5* | 93.80% | 5.60% | 0.30% | 0.30% | 45.00% | 100 | -1.99 |
| *SlHECT6* | 88.20% | 9.90% | 1.00% | 0.80% | 72.51% | 54.6341 | -4.46 |
| *SlHECT7* | 89.80% | 8.90% | 0.80% | 0.50% | 83.65% | 68.84 | -3.52 |
| *SlHECT8* | 82.80% | 12.40% | 1.80% | 2.90% | 35.09% | 55.7143 | -7.72 |
| *SlHECT9* | 91.70% | 6.70% | 0.80% | 0.80% | 78.91% | 67.4074 | -5.16 |
| *SlHECT10* | 88.20% | 10.10% | 1.70% | 0.00% | 45.38% | 85.1852 | -4.27 |
| *SlHECT11* | 81.50% | 13.40% | 2.10% | 3.10% | 36.89% | 60.1449 | -10.2 |
| *SlHECT12* | 89.30% | 9.10% | 0.80% | 0.80% | 82.65% | 57.0025 | -5.14 |
| *SlHECT13* | 89.60% | 8.90% | 0.80% | 0.80% | 68.20% | 64.8352 | -3.1 |
| *SlHECT14* | 88.20% | 9.50% | 1.30% | 1.00% | 75.18% | 62.6506 | -5.41 |

**Supplementary Table S8:** Model validation and quality assessment of tomato HECT gene family.


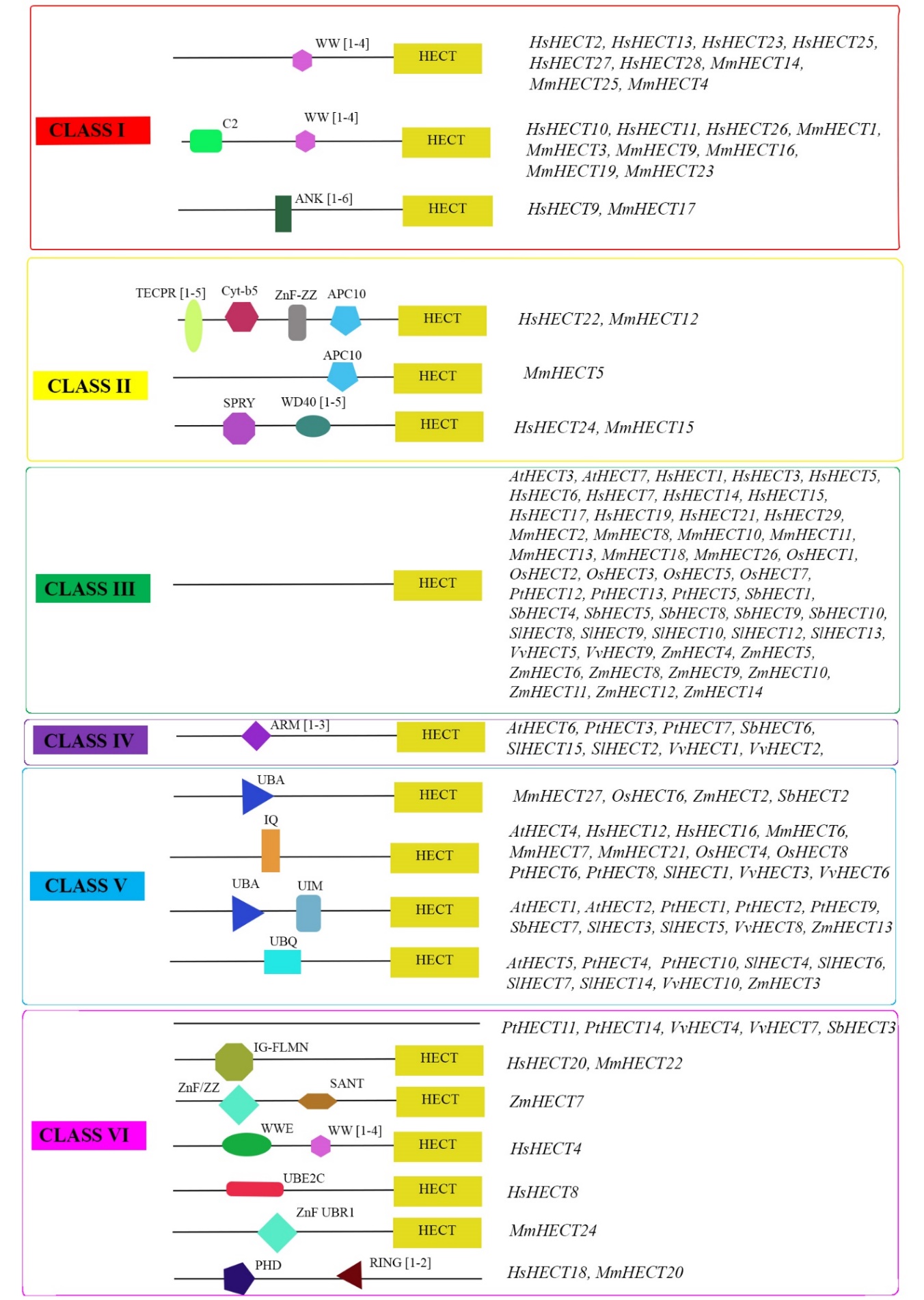


**Supplementary Figure S9:** HECT gene family were classified into six sub-classes based on different domains present at the N-terminal to HECT domain, represented with different colors and shapes, corresponding to Solanum lycopersicum, Oryza Sativa, Zea mays, Vitis vinifera, Sorghum bicolor, Arabidopsis thaliana, Populus trichocarpa, Mus Musculus and Homo sapiens.

**
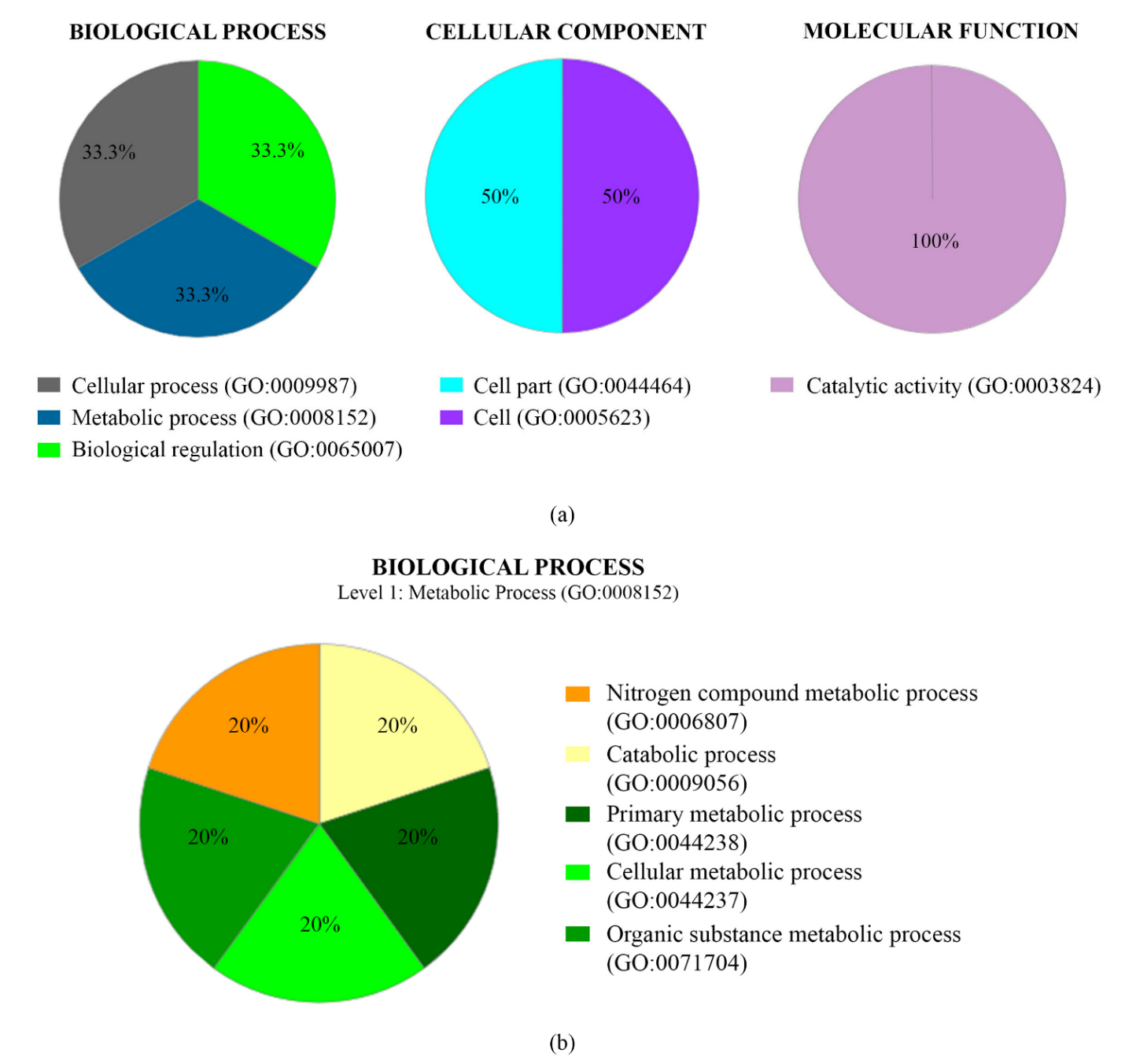
**

**Supplementary Figure S10:** The gene ontology (GO) analysis of the 14 HECT E3 ubiquitin ligases in tomato, depicting (a) their roles in biological process, cellular component and molecular function, respectively, and (b) their involvement in various metabolic processes.

| **Organism** | **Cluster/ Module** | **Number of Nodes** | **Number of Edges** | **Cluster Score** |
| --- | --- | --- | --- | --- |
| ***Solanum lycopersicum*** | 1 | 34 | 93 | 5.455 |
|  | 2 | 6 | 25 | 5.200 |
|  | 3 | 20 | 63 | 5.158 |

**Supplementary Table S11:** Details of clusters of all PPI networks using MCODE plug-in of Cytoscape, describing the number of nodes and edges, and cluster score of each module.

| **GO Category** | **Description** | **Number of genes (Tomato)** |
| --- | --- | --- |
| **MOLECULAR FUNCTION** | Other | 3 |
|  | Anaphase-promoting complex binding | 0 |
|  | Catalytic activity | 0 |
|  | GTP binding | 0 |
|  | Ligand-gated sodium channel activity | 0 |
|  | Lys63-specific deubiquitinase activity | 1 |
|  | Proteasome binding | 0 |
|  | Protein dimerization activity | 0 |
|  | Signaling receptor activity | 0 |
|  | Sodium channel activity | 0 |
|  | Thiol-dependent ubiquitinyl hydrolase activity | 2 |
|  | NEDD8 activating enzyme activity | 0 |
|  | Notch binding | 0 |
|  | DNA binding | 0 |
|  | Growth factor activity | 0 |
|  | Nucleic acid binding | 0 |
|  | Thiol-dependent ubiquitin-specific protease activity | 1 |
|  | ATPase activity | 0 |
|  | Isopeptidase activity | 1 |
|  | Metallopeptidase activity | 1 |
|  | Ubiquitin-ubiquitin ligase activity | 2 |
|  | Proteasome-activating ATPase activity | 0 |
|  | Enzyme regulator activity | 0 |
|  | Protein serine/threonine kinase activity | 0 |
|  | Kinase activity | 0 |
|  | Transmembrane receptor protein serine/threonine kinase activity | 0 |
|  | Calcium ion binding | 0 |
|  | Ubiquitin protein ligase binding | 0 |
|  | Hydrolase activity | 1 |
|  | Metal ion binding | 0 |
|  | Protein kinase activity | 0 |
|  | Structural constituent of ribosome | 7 |
|  | DNA-binding transcription factor activity | 0 |
|  | Endopeptidase activity | 0 |
|  | Ubiquitin-like modifier activating enzyme activity | 0 |
|  | Threonine-type endopeptidase activity | 0 |
|  | ATP binding | 5 |
|  | Ubiquitin protein ligase activity | 0 |
|  | Zinc ion binding | 0 |
|  | Ubiquitin-protein transferase activity | 5 |
|  | Protein binding | 31 |
| **BIOLOGICAL PROCESS** | Others | 4 |
|  | Autophagy | 0 |
|  | Cellular response to DNA damage stimulus | 0 |
|  | Regulation of mitotic metaphase/anaphase transition | 0 |
|  | Sodium ion transport | 0 |
|  | Anaphase-promoting complex-dependent catabolic process | 0 |
|  | Immune system process | 0 |
|  | Protein neddylation | 0 |
|  | Regulation of cell cycle | 0 |
|  | Ribosome-associated ubiquitin-dependent protein catabolic process | 0 |
|  | DNA repair | 1 |
|  | Proteasome assembly | 0 |
|  | Proteasome-mediated ubiquitin-dependent protein catabolic process | 0 |
|  | Protein deubiquitination | 2 |
|  | Ubiquitin-dependent protein catabolic process via the N-end rule pathway | 0 |
|  | Double-strand break repair | 0 |
|  | Regulation of protein catabolic process | 0 |
|  | Cell communication | 0 |
|  | Transmembrane receptor protein serine/threonine kinase signaling pathway | 0 |
|  | Cellular protein modification process | 0 |
|  | Notch signaling pathway | 0 |
|  | Transforming growth factor beta receptor signaling pathway | 0 |
|  | Protein phosphorylation | 0 |
|  | Translation | 7 |
|  | Proteolysis | 0 |
|  | Regulation of transcription, DNA-templated | 1 |
|  | Multicellular organism development | 0 |
|  | Intracellular signal transduction | 0 |
|  | Proteolysis involved in cellular protein catabolic process | 0 |
|  | Protein ubiquitination | 2 |
|  | Protein catabolic process | 1 |
|  | Ubiquitin-dependent protein catabolic process | 3 |
| **CELLULAR COMPONENT** | Others | 0 |
|  | BRISC complex | 1 |
|  | Proteasome core complex, beta-subunit complex | 0 |
|  | RQC complex | 0 |
|  | BRCA1-A complex | 1 |
|  | Proteasome regulatory particle | 0 |
|  | Proteasome activator complex | 0 |
|  | Cullin-RING ubiquitin ligase complex | 0 |
|  | Nucleus | 1 |
|  | Proteasome complex | 0 |
|  | Ubiquitin ligase complex | 1 |
|  | Anaphase-promoting complex | 0 |
|  | Host cell nucleus | 0 |
|  | Transcription regulator complex | 0 |
|  | Integral component of membrane | 0 |
|  | Proteasome core complex, alpha-subunit complex | 0 |
|  | Ribosome | 7 |
|  | Cytoplasm | 2 |
|  | Membrane | 0 |
|  | Proteasome core complex | 0 |
|  | Others | 0 |
|  | BRISC complex | 1 |
|  | Proteasome core complex, beta-subunit complex | 0 |
|  | RQC complex | 0 |
|  | BRCA1-A complex | 1 |
|  | Proteasome regulatory particle | 0 |
|  | Proteasome activator complex | 0 |
|  | Cullin-RING ubiquitin ligase complex | 0 |
|  | Nucleus | 1 |
|  | Proteasome complex | 0 |
|  | Ubiquitin ligase complex | 1 |
|  | Anaphase-promoting complex | 0 |
|  | Host cell nucleus | 0 |
|  | Transcription regulator complex | 0 |
|  | Integral component of membrane | 0 |
|  | Proteasome core complex, alpha-subunit complex | 0 |
|  | Ribosome | 7 |
|  | Cytoplasm | 2 |
|  | Membrane | 0 |
|  | Proteasome core complex | 0 |

**Supplementary Table S12:** GO-based functional analysis of HECT E3 ligases and interacting partners from tomato.

| **CATEGORY** | **PATHWAY** | **PATHWAY ID** | **NUMBER OF GENES** | **KO IDENTITIES** |
| --- | --- | --- | --- | --- |
| ***Solanum lycopersicum*** | | | | |
| **Genetic Information Processing** | Protein processing in endoplasmic reticulum | 4141 | 3 | K06689, K10597, K02927, K10589, K10590 |
|  | Ubiquitin mediated proteolysis | 04120 | 25 | K02977, K04551, K04649, K06689, K08770, K10580, K10597 |
|  | Proteasome | 3050 | 7 | K03029, K03030, K03036, K03066, K06691 |
|  | Homologous recombination | 03440 | 1 | K11864 |
|  | Ribosome | 3010 | 8 | K02977, K02927 |
|  | Fanconi anemia pathway | 3460 | 1 | K13960 |
| **Environmental Information Processing** | MAPK signaling pathway - fly | 4013 | 3 | K06689 |
| **Cellular Processes** | Mitophagy - yeast | 04139 | 2 | K11841 |
|  | Mitophagy - animal | 4137 | 13 | K02977, K04551, K08770, K02927 |
| **Organismal Systems** | Toll and Imd signaling pathway | 4624 | 5 | K06689, K10580 |
|  | NOD-like receptor signaling pathway | 4621 | 1 | K11864 |
|  | PPAR signaling pathway | 03320 | 3 | K08770 |
| **Human Diseases** | Alzheimer disease | 05010 | 7 | K03029, K03030, K03036, K03066, K06691 |
|  | Parkinson disease | 05012 | 21 | K02977, K03029, K03030, K03036, K03066, K04551, K06691, K08770, K02927 |
|  | Huntington disease | 5016 | 7 | K03029, K03030, K03036, K03066, K06691, K02927 |
|  | Spinocerebellar ataxia | 05017 | 12 | K03029, K03030, K03036, K03066, K06691 |
|  | Shigellosis | 05131 | 12 | K02977, K04551, K06689, K08770, K10580 |
|  | Kaposi sarcoma-associated herpesvirus infection | 05167 | 14 | K02977, K04551, K08770, K02927 |
|  | Epstein-Barr virus infection | 05169 | 7 | K03029, K03030, K03036, K03066, K06691 |
|  | Viral carcinogenesis | 5203 | 6 | K11643 |
|  | Human papillomavirus infection | 5165 | 6 | K11644 |

**Supplementary Table S13:** Pathway analysis data of all the HECT genes and its interactors from tomato, depicting their roles in different biological pathways.


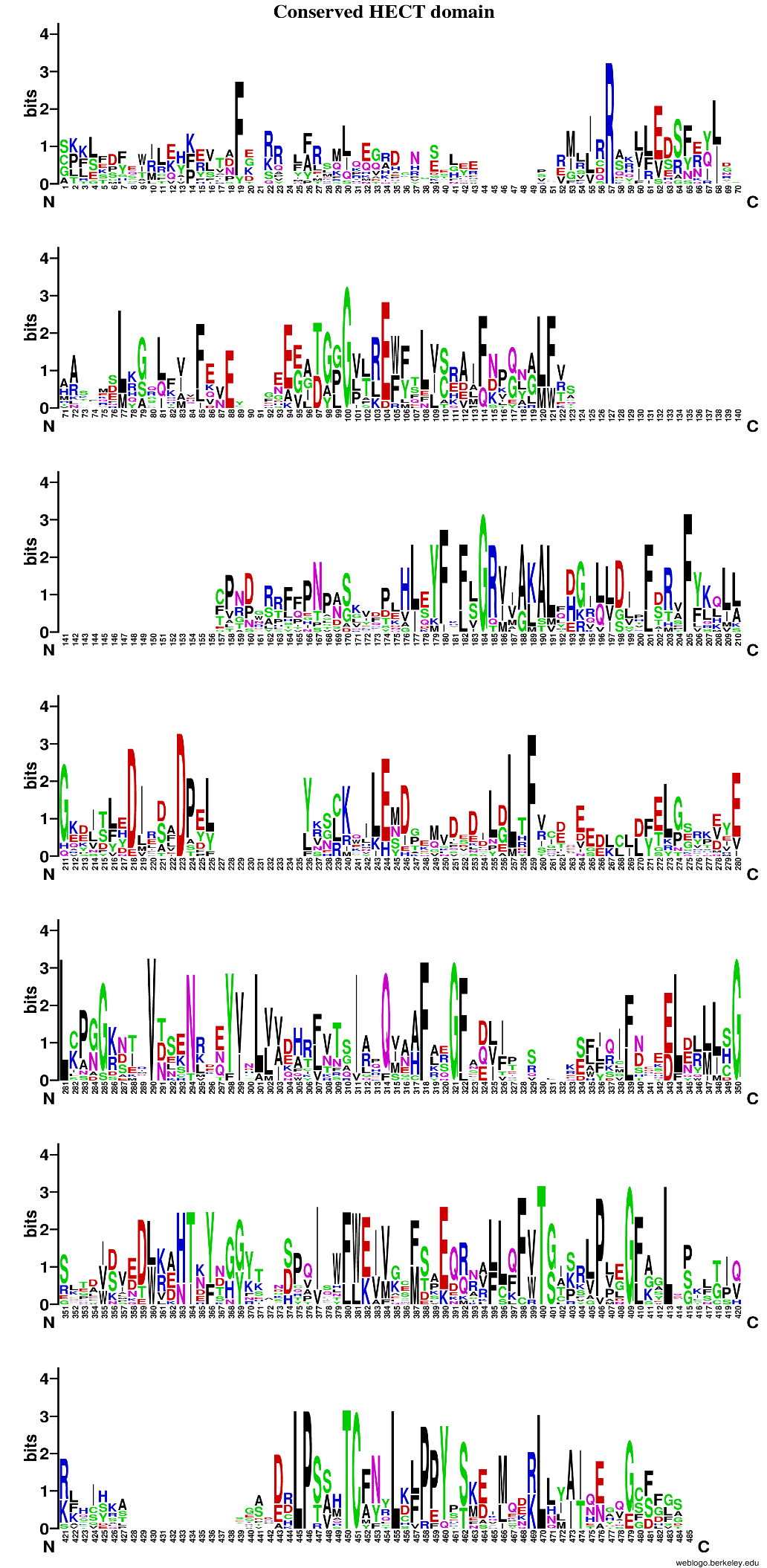


**Supplementary Figure** **S14**: Logo of conserved residues in HECT domain of Solanum lycopersicum.

| **Identifier** | **Species** | **Strain** | **Cultivar** | **Organ Type** | **Organ** | **Tissue** | **Stage** |
| --- | --- | --- | --- | --- | --- | --- | --- |
| **C1** | S lyco | Wild Type | Heinz.1706 | Vegetative | Root | Whole | NC.(root) |
| **C2** | S lyco | Wild Type | Heinz.1706 | Vegetative | Leaf | Whole | NC.(leaf) |
| **C3** | S lyco | Wild Type | Heinz.1706 | Reproductive | Flower | Whole | NC.(flower.bud) |
| **C4** | S lyco | Wild Type | Heinz.1706 | Reproductive | Flower | Whole | NC.(flower) |
| **C5** | S lyco | Wild Type | Heinz.1706 | Reproductive | Fruit | Whole | 1.cm |
| **C6** | S lyco | Wild Type | Heinz.1706 | Reproductive | Fruit | Whole | 2.cm |
| **C7** | S lyco | Wild Type | Heinz.1706 | Reproductive | Fruit | Whole | 3.cm |
| **C8** | S lyco | Wild Type | Heinz.1706 | Reproductive | Fruit | Whole | Mature.Green |
| **C9** | S lyco | Wild Type | Heinz.1706 | Reproductive | Fruit | Whole | Breaker |
| **C10** | S lyco | Wild Type | Heinz.1706 | Reproductive | Fruit | Whole | Breaker.10 |

**Supplementary Table S15:** Gene expression conditions for vegetative and reproductive tissues.

| **Identifier** | **Species** | **Strain** | **Cultivar** | **Organ Type** | **Organ** | **Tissue** | **Stage** | **Treatment** | **Duration** | **Pathogen** |
| --- | --- | --- | --- | --- | --- | --- | --- | --- | --- | --- |
| **C1** | S lyco | Wild Type | M82 | Vegetative | Seedling | Whole | 10.dpg | sun | - | - |
| **C2** | S lyco | Wild Type | M82 | Vegetative | Seedling | Whole | 10.dpg | shade | - | - |
| **C3** | S lyco | Wild Type | M82 | Vegetative | Seedling | Whole | 10.dpg | shade | - | - |
| **C4** | S lyco | Wild Type | M82 | Vegetative | Seedling | Leaf | 2.young.leaves | PAC | 3d | - |
| **C5** | S lyco | Wild Type | M82 | Vegetative | Seedling | Leaf | 2.young.leaves | PAC_GA | 3d_30min | - |
| **C6** | S lyco | Wild Type | M82 | Vegetative | Root | Whole | 10.dpg | sun | - | - |
| **C7** | S lyco | Wild Type | M82 | Vegetative | Root | Whole | 10.dpg | shade | - | - |
| **C8** | S lyco | Wild Type | Avigail.(870) | Vegetative | Root | Whole | 15.dpg._0dpi | Meljav | 0d | *Meloidogyne.javanica* |
| **C9** | S lyco | Wild Type | Avigail.(870) | Vegetative | Root | Whole | 15.dpg._2dpi | Meljav | 2d | *Meloidogyne.javanica* |
| **C10** | S lyco | Wild Type | Avigail.(870) | Vegetative | Root | Whole | 15.dpg._5dpi | Meljav | 5d | *Meloidogyne.javanica* |
| **C11** | S lyco | Wild Type | Avigail.(870) | Vegetative | Root | Whole | 15.dpg._15dpi | Meljav | 15d | *Meloidogyne.javanica* |
| **C12** | S lyco | Wild Type | M82 | Vegetative | Meristem | Shoot.Apical.Meristem | 19.DAPL | Shade | Cont | - |
| **C13** | S lyco | Wild Type | M82 | Vegetative | Meristem | Shoot.Apical.Meristem | 19.DAPL | Sun | Cont | - |
| **C14** | S lyco | Wild Type | M82 | Vegetative | Meristem | Shoot.Apical.Meristem | 19.DAPL | Tr Shade | 28 h | - |
| **C15** | S lyco | Wild Type | M82 | Vegetative | Meristem | Shoot.Apical.Meristem | 19.DAPL | CTRL Sun Tr Shade | Cont | - |
| **C16** | S lyco | Wild Type | M82 | Vegetative | Meristem | Whole | 35.dpg | sun | - | - |
| **C17** | S lyco | Wild Type | M82 | Vegetative | Meristem | Whole | 35.dpg | shade | - | - |
| **C18** | S lyco | Wild Type | M82 | Vegetative | Stem | Whole | 50.dpg | shade | - | - |
| **C19** | S lyco | Wild Type | M82 | Vegetative | Stem | Whole | 50.dpg | sun | - | - |
| **C20** | S lyco | Wild Type | Ailsa.Craig | Vegetative | Leaf | Whole | nc | VIGS_AGO1 | 3h | *Agrobacterium.tumefaciens.GV3101* |
| **C21** | S lyco | Wild Type | Ailsa.Craig | Vegetative | Leaf | Whole | nc | VIGS_Ctrl | 3h | *Agrobacterium.tumefaciens.GV3101* |
| **C22** | S lyco | Wild Type | M82 | Vegetative | Leaf | Leaf.primordium | 19.DAPL | Shade | Cont | *-* |
| **C23** | S lyco | Wild Type | M82 | Vegetative | Leaf | Leaf.primordium | 19.DAPL | Sun | Cont | *-* |
| **C24** | S lyco | Wild Type | M82 | Vegetative | Leaf | Leaf.primordium | 19.DAPL | Tr Shade | 28 h | *-* |
| **C25** | S lyco | Wild Type | M82 | Vegetative | Leaf | Leaf.primordium | 19.DAPL | Sun Tr shift | Cont | *-* |
| **C26** | S lyco | Wild Type | TYLCV-resistant.CLN2777A | Vegetative | Leaf | Whole | 2.leaves | TYLC | 0d | *Tomato.yellow.leaf.curl.virus* |
| **C27** | S lyco | Wild Type | TYLCV-resistant.CLN2777A | Vegetative | Leaf | Whole | 2.leaves | TYLC | 3-7d | *Tomato.yellow.leaf.curl.virus* |
| **C28** | S lyco | Wild Type | TYLCV-susceptible.TMXA48-4-0 | Vegetative | Leaf | Whole | 2.leaves | TYLC | 0d | *Tomato.yellow.leaf.curl.virus* |
| **C29** | S lyco | Wild Type | TYLCV-susceptible.TMXA48-4-0 | Vegetative | Leaf | Whole | 2.leaves | TYLC | 3-7d | *Tomato.yellow.leaf.curl.virus* |
| **C30** | S lyco | Wild Type | M82 | Vegetative | Leaf | Whole | 50.dpg | sun | - | - |
| **C31** | S lyco | Wild Type | M82 | Vegetative | Leaf | Whole | 50.dpg | shade | - | - |
| **C32** | S lyco | Wild Type | Moneymaker | Vegetative | Leaf | Whole | 56.DAPL | Ctrl | - | - |
| **C33** | S lyco | Wild Type | Moneymaker | Vegetative | Leaf | Whole | 56.DAPL | HS_2 | 1h_39°C | - |
| **C34** | S lyco | Wild Type | M82 | Reproductive | Flower | Whole | 50.dpg | sun | - | - |
| **C35** | S lyco | Wild Type | M82 | Reproductive | Flower | Whole | 50.dpg | shade | - | - |
| **C36** | S lyco | Wild Type | Moneymaker | Reproductive | Flower | Anthers | 56.DAPL | Ctrl | - | - |
| **C37** | S lyco | Wild Type | Moneymaker | Reproductive | Flower | Anthers | 56.DAPL | HS_2 | 1h_39°C | - |
| **C38** | S lyco | Wild Type | M82 | Reproductive | Fruit | Whole | IG+MG | sun | - | - |
| **C39** | S lyco | Wild Type | M82 | Reproductive | Fruit | Whole | IG+MG | shade | - | - |
| **C40** | S lyco | Wild Type | Moneymaker | Reproductive | Fruit | Whole | Breaker | Ctrl | - | - |
| **C41** | S lyco | Wild Type | Moneymaker | Reproductive | Fruit | Whole | Breaker | Fu_mos | - | *Funneliformis.mosseae* |

**Supplementary Table S16**: Gene expression conditions for biotic and abiotic stress treatments.

| **Identifier** | **Species** | **Strain** | **Cultivar** | **Organ Type** | **Organ** | **Tissue** | **Stage** | **Treatment** | **Duration** |
| --- | --- | --- | --- | --- | --- | --- | --- | --- | --- |
| **C1** | S lyco | Wild Type | Micro.Tom | Vegetative | Root | Whole | 14.dpg | DMSO | 24h |
| **C2** | S lyco | Wild Type | Micro.Tom | Vegetative | Root | Whole | 14.dpg | Cytok | 24h |
| **C3** | S lyco | Wild Type | Micro.Tom | Vegetative | Root | Whole | 14.dpg | Auxin | 24h |
| **C4** | S lyco | Wild Type | Micro.Tom | Vegetative | Root | Lateral | 14.dpg | DMSO | 24h |
| **C5** | S lyco | Wild Type | Micro.Tom | Vegetative | Root | Lateral | 14.dpg | Cytok | 24h |
| **C6** | S lyco | Wild Type | Micro.Tom | Vegetative | Root | Lateral | 14.dpg | Auxin | 24h |
| **C7** | S lyco | Wild Type | Micro.Tom | Vegetative | Root | Tip | 14.dpg | DMSO | 24h |
| **C8** | S lyco | Wild Type | Micro.Tom | Vegetative | Root | Tip | 14.dpg | Cytok | 24h |
| **C9** | S lyco | Wild Type | Micro.Tom | Vegetative | Root | Tip | 14.dpg | Auxin | 24h |
| **C10** | S lyco | Wild Type | Micro.Tom | Vegetative | Leaf | Whole | 13.dpg | DMSO | 2h |
| **C11** | S lyco | Wild Type | Micro.Tom | Vegetative | Leaf | Whole | 13.dpg | Cytok | 2h |
| **C12** | S lyco | Wild Type | Micro.Tom | Vegetative | Leaf | Whole | 13.dpg | DMSO | 24h |
| **C13** | S lyco | Wild Type | Micro.Tom | Vegetative | Leaf | Whole | 13.dpg | Cytok | 24h |
| **C14** | S lyco | Wild Type | Micro.Tom | Vegetative | Leaf | Whole | 35.dpg | DMSO | 2h |
| **C15** | S lyco | Wild Type | Micro.Tom | Vegetative | Leaf | Whole | 35.dpg | Cytok | 2h |
| **C16** | S lyco | Wild Type | Micro.Tom | Vegetative | Leaf | Whole | 35.dpg | DMSO | 24h |
| **C17** | S lyco | Wild Type | Micro.Tom | Vegetative | Leaf | Whole | 35.dpg | Cytok | 24h |
| **C18** | S lyco | Wild Type | Micro.Tom | Reproductive | Fruit | Whole | Mature.Green | Ctrl | 0h |
| **C19** | S lyco | Wild Type | Micro.Tom | Reproductive | Fruit | Whole | Mature.Green | Ctrl | 48h |
| **C20** | S lyco | Wild Type | Micro.Tom | Reproductive | Fruit | Whole | Mature.Green | IAA | 48h |
| **C21** | S lyco | Wild Type | Micro.Tom | Reproductive | Fruit | Whole | Mature.Green | ACC | 48h |
| **C22** | S lyco | Wild Type | Micro.Tom | Reproductive | Fruit | Whole | Mature.Green | IAA+ACC | 48h |

**Supplementary Table S17**: Gene expression conditions for hormonal stress treatments.

| **Motif**  **No.** | **Accession** | **Superfamily** | **Description** | **Gene Ontology** | | |
| --- | --- | --- | --- | --- | --- | --- |
|  |  |  |  | **Biological Process** | **Cellular Component** | **Molecular function** |
| 1 | cl27008 | HECTc | C-terminal catalytic domain of a subclass of Ubiquitin-protein ligase (E3) | protein ubiquitination (GO:0016567);  response to toxic substance (GO:0009636);  leaf senescence (GO:0010150);  response to hydrogen peroxide (GO:0042542);  response to antibiotic (GO:0046677);  response to drug (GO:0042493);  response to jasmonic acid (GO:0009753);  response to acid chemical (GO:000110) | Cytoplasm (GO:0005737) | Ubiquitin protein ligase activity (GO:0061630) |
| 2 | cl27008 | HECTc |  |  |  |  |
| 3 | cl27008 | HECTc |  |  |  |  |
| 4 | cl27008 | HECTc |  |  |  |  |
| 5 | cl27008 | HECTc |  |  |  |  |
| 7 | cl27008 | HECTc |  |  |  |  |
| 8 | -- | -- |  |  |  |  |
| 10 | cl27008 | HECTc |  | -- | -- | -- |
| 9 | -- | -- | -- | Cellular component morphogenesis (GO:0032989);  protein ubiquitination (GO:0016567);  DNA metabolic process (GO:0006259);  DNA endoreduplication (GO:0042023);  trichome branching (GO:0010091) | Plasma membrane (GO:0005886);  Cytoplasm (GO:0005737) | Ubiquitin protein ligase activity (GO:0061630) |
| 6 | cl34886 | SRP1 | Karyopherin (importin) alpha [Intracellular trafficking and secretion] |  |  |  |

**Supplementary Table S18:** Predicted biological functions of the 10 novel motifs identified in tomato HECT gene family from MEME suite, using Conserved Domains Database (CDD) and EggNOG mapper tool.
